# Coronary Artery and Cardiac Disease in Patients With Type 2 Myocardial Infarction: A Prospective Cohort Study

**DOI:** 10.1161/CIRCULATIONAHA.121.058542

**Published:** 2022-03-28

**Authors:** Anda Bularga, John Hung, Marwa Daghem, Stacey Stewart, Caelan Taggart, Ryan Wereski, Trisha Singh, Mohammed N. Meah, Takeshi Fujisawa, Amy V. Ferry, Justin Chiong, William S. Jenkins, Fiona E. Strachan, Scott Semple, Edwin J.R. van Beek, Michelle Williams, Damini Dey, Chris Tuck, Andrew H. Baker, David E. Newby, Marc R. Dweck, Nicholas L. Mills, Andrew R. Chapman

**Affiliations:** 1 BHF Centre for Cardiovascular Science (A.B., J.H., M.D., S.S., C.T., R.W., T.S., M.N.M., T.F., A.V.F., J.C., W.S.J., F.E.S., M.W., C.T., A.H.B., D.E.N., M.R.D., N.L.M., A.R.C.), University of Edinburgh, United Kingdom.; 2 Edinburgh Imaging (S.S., E.J.R.v.B., M.W.), University of Edinburgh, United Kingdom.; 3 Usher Institute (N.L.M.), University of Edinburgh, United Kingdom.; 4 Biomedical Imaging Research Institute, Cedars-Sinai Medical Center, Los Angeles, CA (D.D.).

**Keywords:** cardiac imaging techniques, echocardiography, magnetic resonance imaging, myocardial infarction

## Abstract

Type 2 myocardial infarction is caused by myocardial oxygen supply-demand imbalance, and its diagnosis is increasingly common with the advent of high-sensitivity cardiac troponin assays. Although this diagnosis is associated with poor outcomes, widespread uncertainty and confusion remain among clinicians as to how to investigate and manage this heterogeneous group of patients with type 2 myocardial infarction.

**Methods:**

In a prospective cohort study, 8064 consecutive patients with increased cardiac troponin concentrations were screened to identify patients with type 2 myocardial infarction. We excluded patients with frailty or renal or hepatic failure. All study participants underwent coronary (invasive or computed tomography angiography) and cardiac (magnetic resonance or echocardiography) imaging, and the underlying causes of infarction were independently adjudicated. The primary outcome was the prevalence of coronary artery disease.

**Results:**

In 100 patients with a provisional diagnosis of type 2 myocardial infarction (median age, 65 years [interquartile range, 55–74 years]; 43% women), coronary and cardiac imaging reclassified the diagnosis in 7 patients: type 1 or 4b myocardial infarction in 5 and acute myocardial injury in 2 patients. In those with type 2 myocardial infarction, median cardiac troponin I concentrations were 195 ng/L (interquartile range, 62–760 ng/L) at presentation and 1165 ng/L (interquartile range, 277–3782 ng/L) on repeat testing. The prevalence of coronary artery disease was 68% (63 of 93), which was obstructive in 30% (28 of 93). Infarct-pattern late gadolinium enhancement or regional wall motion abnormalities were observed in 42% (39 of 93), and left ventricular systolic dysfunction was seen in 34% (32 of 93). Only 10 patients had both normal coronary and normal cardiac imaging. Coronary artery disease and left ventricular systolic dysfunction were previously unrecognized in 60% (38 of 63) and 84% (27 of 32), respectively, with only 33% (21 of 63) and 19% (6 of 32) on evidence-based treatments.

**Conclusions:**

Systematic coronary and cardiac imaging of patients with type 2 myocardial infarction identified coronary artery disease in two-thirds and left ventricular systolic dysfunction in one-third of patients. Unrecognized and untreated coronary or cardiac disease is seen in most patients with type 2 myocardial infarction, presenting opportunities for initiation of evidence-based treatments with major potential to improve clinical outcomes.

**Registration:**

URL: https://www.clinicaltrials.gov; Unique identifier: NCT03338504.

**Clinical Perspective**

**What Is New?**

- DEMAND-MI (Determining the Mechanism of Myocardial Injury and Role of Coronary Disease in Type 2 Myocardial Infarction) is the first prospective study to undertake systematic cardiac imaging in 100 patients with type 2 myocardial infarction.
- Cardiac imaging led to reclassification of the diagnosis in 7 of 100 patients.
- In those with confirmed type 2 myocardial infarction, two-thirds had coronary artery disease, and one-third had left ventricular impairment, which were previously unrecognized and untreated in the majority.
- Fewer than half of all patients with an adjudicated diagnosis of type 2 myocardial infarction had myocardial scar or regional wall motion abnormality on imaging, usually associated with myocardial infarction.

**What Are the Clinical Implications?**

- In patients with type 2 myocardial infarction, investigation with invasive or noninvasive coronary and cardiac imaging should be considered because the identification of unrecognized coronary artery disease and left ventricular impairment will have immediate and long-term implications for treatment.
- In those patients without imaging evidence of myocardial infarction or coronary artery disease, it is unlikely that the patient will benefit from therapies targeting coronary atherosclerosis; in this setting, the value of a diagnosis of type 2 myocardial infarction is questionable.

**Editorial, see p 1201**

In 2007, the Universal Definition of Myocardial Infarction introduced a classification of myocardial infarction according to pathogenesis.^1^ Type 1 myocardial infarction is defined as a myocardial infarction that occurs as a result of thrombosis associated with atherosclerotic plaque.^1^ In this setting, there are established evidence-based strategies for investigation and treatment that improve outcomes.^2,3^ In contrast, type 2 myocardial infarction results from an imbalance in myocardial oxygen supply or demand without atherothrombosis.^1,4,5^ It encompasses a diverse and heterogeneous group of patients who present with disturbed physiology in the context of an acute illness such as tachycardia, hypoxemia, or hypotension or in those with a coronary mechanism other than atherothrombosis such as coronary embolism or coronary artery dissection.^4,6^ The definition of type 2 myocardial infarction is based on expert consensus, without prospective evidence to support the benefits of such a diagnosis for the management or treatment of these patients. It is now recognized that half of all cardiac troponin elevations are attributable to either type 2 myocardial infarction or myocardial injury,^7,8^ especially in the era of high-sensitivity cardiac troponin assays.^8,9^ However, for such a prevalent condition, our understanding of the underlying disease mechanisms remains limited.^4^ Fewer than one-third of patients with type 2 myocardial infarction are managed by cardiologists,^10^ and just 10% to 20% undergo investigations to identify the presence of underlying coronary artery or cardiac disease.^10–12^

Through a prospective cohort study, we aimed to evaluate the prevalence of coronary artery and cardiac disease in patients with type 2 myocardial infarction with systematic coronary and cardiac imaging to better understand the pathogenesis of this condition and to identify potential treatment opportunities.

## Methods

### Transparency and Openness Promotion

As an educational resource, anonymized data for all participants, including a summary of the clinical presentation and all study investigations, are publicly available in an online repository that can be accessed after registration.^13^ We conducted a data protection impact assessment, which was approved by the University of Edinburgh’s data protection officer (Supplemental Material). Summary data can be made available on request from the corresponding author.

### Study Design and Oversight

DEMAND-MI (Determining the Mechanism of Myocardial Injury and Role of Coronary Disease in Type 2 Myocardial Infarction) is a prospective observational cohort study. The study was registered on ClinicalTrials.gov (Unique identifier: NCT03338504) and approved by the South East Scotland Regional Ethics Committee (17/SS/0078), the Academic and Clinical Central Office for Research and Development, and the NHS Lothian Health Board. The study was conducted in accordance with the Declaration of Helsinki with written informed consent of all participants. The Caldicott Guardian approved data linkage to enable screening of consecutive patients.

### Patient Population

All patients attending the Royal Infirmary of Edinburgh, Scotland, for whom cardiac troponin was requested by the attending clinician were screened with a tool embedded in the electronic patient record.^14,15^ Cardiac troponin concentrations were measured with the ARCHITECT_*STAT*_ high-sensitivity cardiac troponin I assay (Abbott Laboratories, Abbott Park, IL). This assay has a limit of detection of 1.2 ng/L and an interassay coefficient of variation of <10% at 4.7 ng/L.^16^ The sex-specific upper reference limit or 99th centile is 16 ng/L in women and 34 ng/L in men.^17^ The electronic patient record was reviewed in all patients with an elevated troponin concentration to identify those who met the criteria for a clinical diagnosis of type 2 myocardial infarction. Patients with evidence of acute myocardial injury, defined as a rise or fall in plasma high-sensitivity cardiac troponin I concentration with at least 1 value above the sex-specific 99th centile, and symptoms or signs of myocardial ischemia on the 12-lead ECG in whom there was objective evidence of myocardial oxygen supply or demand imbalance were eligible (Supplemental Material). We did not recruit patients who were unable or unwilling to provide informed consent, those in whom the responsible clinician suspected type 1 myocardial infarction, women who were pregnant or breastfeeding, those with renal impairment (estimated glomerular filtration rate ≤30 mL·min^−1^·1.73 m^−2^) or severe hepatic impairment, or those with advanced frailty and inability to self-transfer (determined with the Katz index)^18^ in whom it would not be feasible or appropriate to perform invasive or extended study procedures.

### Study Procedures

Complete details on the trial and imaging protocols including image analysis are available in the Supplemental Material.

### Coronary Imaging

Coronary angiography was performed by invasive catheterization or computed tomography (CT), depending on comorbidities and patient preference, in a discussion with the usual care physician (Supplemental Material). In patients with ≥1 stenoses in a major epicardial vessel, fractional flow reserve and optical coherence tomography were performed when possible (Supplemental Material). Coronary CT angiography was performed with a 128-multidetector row CT scanner (Siemens Biograph, Siemens AG, Healthcare Sector, Erlangen, Germany) according to Society of Cardiovascular Computed Tomography guidelines.^19^

### Cardiac Imaging

Cardiac magnetic resonance imaging (MRI) was performed using a 3-T scanner (MAGNETOM Verio, Siemens AG, Healthcare Sector). In a subset of patients who had contraindications to cardiac MRI or were unable to undergo the scan, transthoracic echocardiography was performed according to national guidelines.^20^

### Study Outcomes

The primary outcome was the prevalence of obstructive (stenosis >50% in the left main stem or >70% in a major epicardial vessel) or nonobstructive (evidence of plaque disease and luminal stenosis ≤70%) atherosclerotic coronary artery disease. Secondary outcomes included an assessment of left ventricular systolic function and the pattern of myocardial injury on cardiac imaging. Left ventricular function was defined as normal (ejection fraction ≥55%), mild (ejection fraction >45% and <55%), moderate (ejection fraction >35% and ≤45%), or severe (ejection fraction ≤35%) impairment.^20^ The presence of myocardial infarction was defined as evidence of infarct-pattern late gadolinium enhancement on cardiac MRI or a discrete regional wall motion abnormality in a coronary distribution on echocardiography.

### Diagnostic Adjudication

In all participants, a detailed review of electronic health care records was undertaken by an adjudication panel (A.B., J.H., M.R.D., N.L.M., A.R.C.) with expertise in cardiology, coronary intervention, and cardiac imaging. The panel had access to all clinical data, including laboratory results such as serial cardiac troponin measurements, 12-lead ECG, and study imaging. The final diagnosis was adjudicated by consensus in line with recommendations of the Fourth Universal Definition of Myocardial Infarction.^4^ The diagnosis of type 2 myocardial infarction was confirmed in those with acute myocardial injury and symptoms or signs of myocardial ischemia on the ECG when there was objective evidence of myocardial oxygen supply or demand imbalance and atherothrombosis was excluded on review of all cardiac imaging. Patients found to have evidence of atherosclerotic plaque rupture and thrombosis were reclassified as having type 1 or type 4b myocardial infarction, and those with evidence of nonischemic myocardial injury were reclassified as having acute myocardial injury. The adjudication panel was asked to record (1) the likely cause of type 2 myocardial infarction, including coronary, systemic, and arrhythmic causes (Supplemental Material); (2) the presence of obstructive or nonobstructive coronary artery disease; (3) the presence of left ventricular systolic dysfunction; (4) the presence of structural heart disease; and (5) whether coronary or cardiac imaging had influenced clinical management.

### Statistical Analysis

Baseline characteristics are summarized for the study population. Categorical baseline variables were presented as number (percent). Continuous data are presented as mean±SD or median (interquartile range) according to distribution assessed with the Shapiro-Wilk normality test. We compared baseline characteristics and admission variables in patients according to coronary artery disease status. Group-wise comparisons were performed with Fisher exact, χ^2^, Kruskal-Wallis, or 1-way ANOVA tests as appropriate. All analyses were performed in R (version 3.5.1).

### Role of the Funding Source

The funders played no role in the study design; in the collection, analysis, and interpretation of the data; in the writing of the report; or in the decision to submit the manuscript for publication. The corresponding author had full access to all the data in the study and had final responsibility for the decision to submit for publication.

## Results

### Study Population

**Figure 1.**
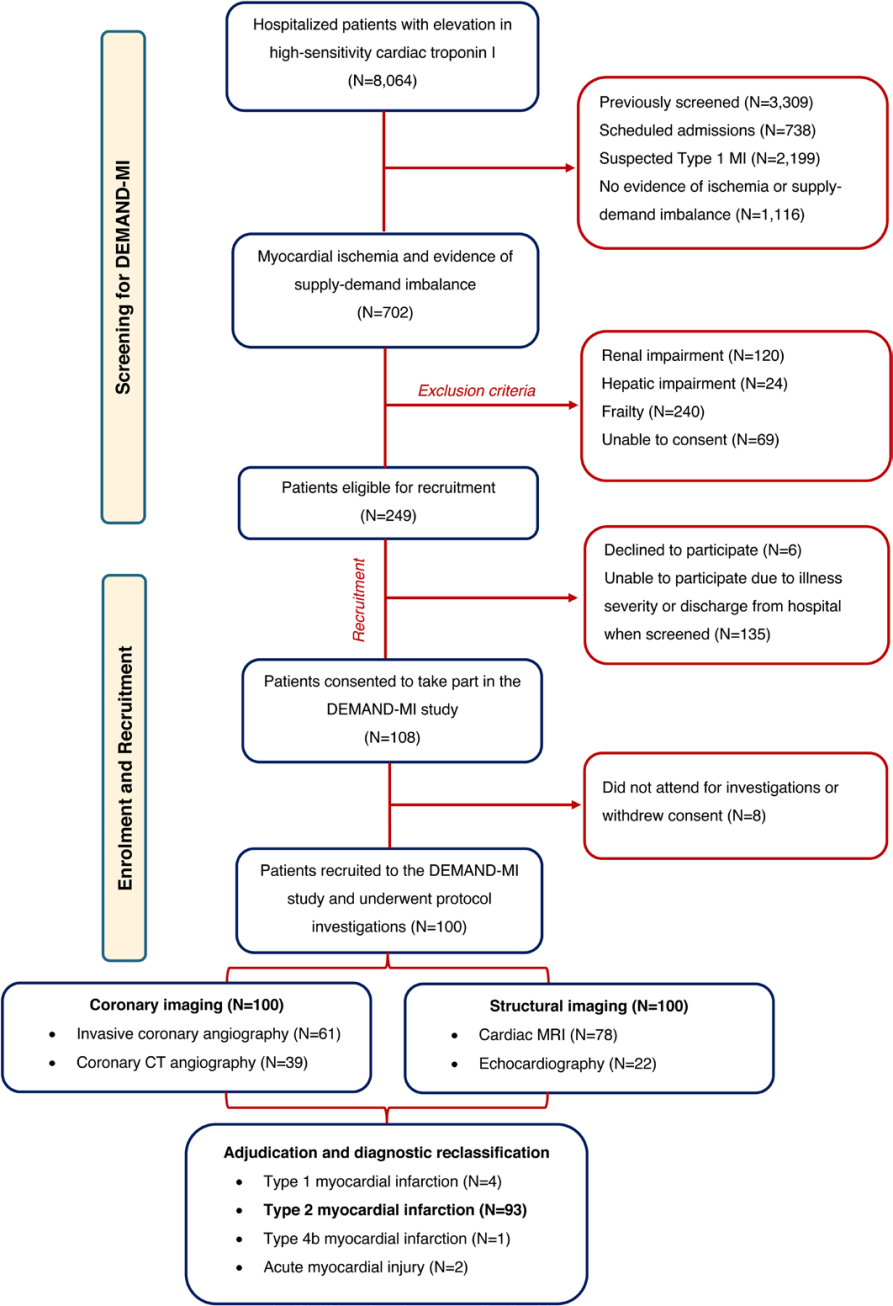
**Study population.** Screening, enrollment, recruitment and final study population with an adjudicated diagnosis of type 2 myocardial infarction. CT indicates computed tomography; DEMAND-MI, Determining the Mechanism of Myocardial Injury and Role of Coronary Disease in Type 2 Myocardial Infarction; and MRI, magnetic resonance imaging.

Between January 2018 and October 2020, 8064 patients with elevated high-sensitivity cardiac troponin I concentrations were screened, of whom 702 (9%) met the diagnostic criteria for type 2 myocardial infarction (Figure 1). After review, 453 patients were found to have ≥1 exclusion criteria. Of 249 patients eligible for recruitment, 135 were unable to participate because of illness severity or discharge from hospital, and 6 declined (Figure 1). A total of 108 patients were enrolled, of whom 100 underwent coronary angiography as part of the study protocol (median age, 65 years [55–74 years]; 43% women; Table 1).

**Table 1.**
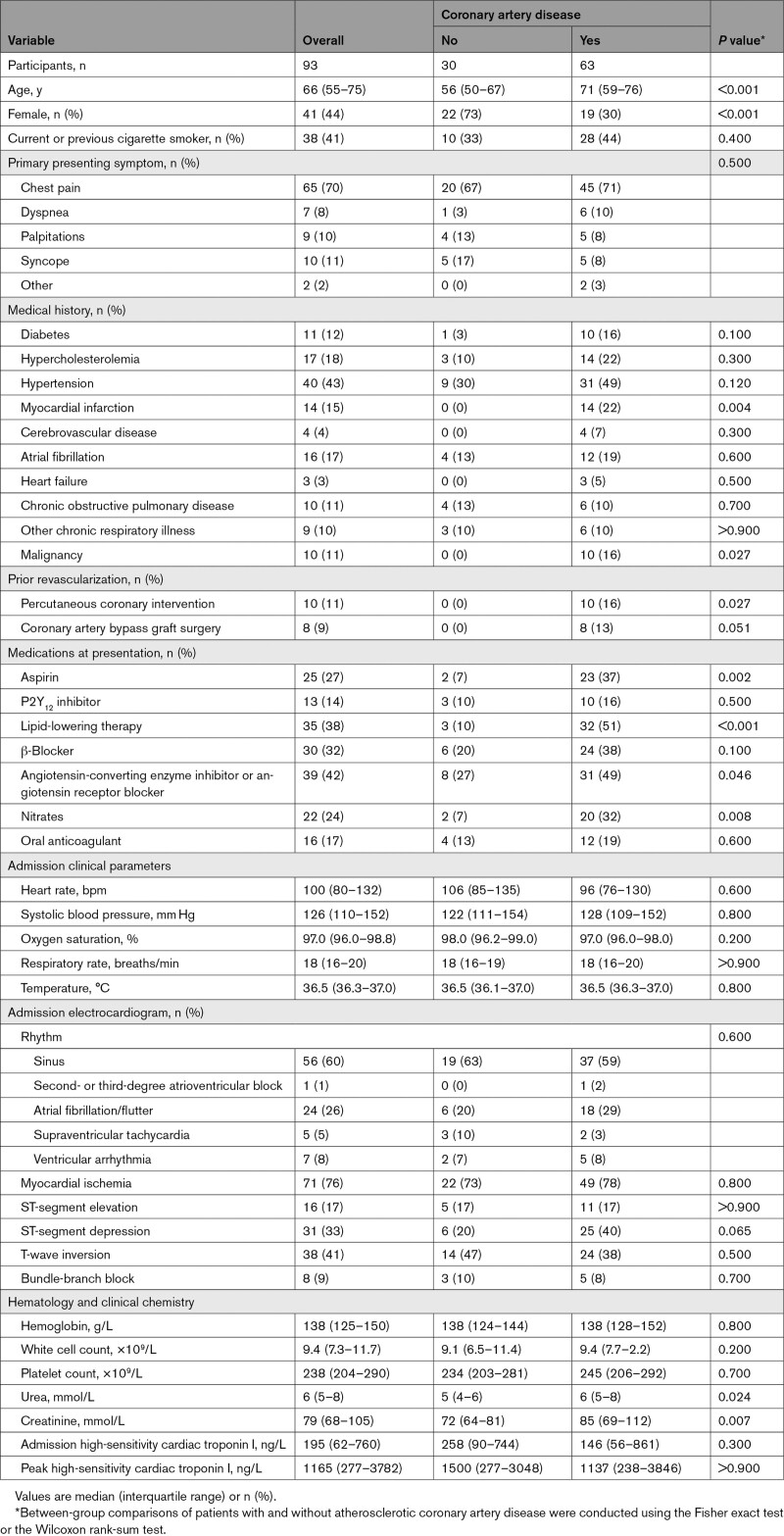
Baseline Characteristics and Admission Parameters for Study Participants With an Adjudicated Diagnosis of Type 2 Myocardial Infarction According to Presence or Absence of Atherosclerotic Coronary Artery Disease on Imaging

**Figure 2.**
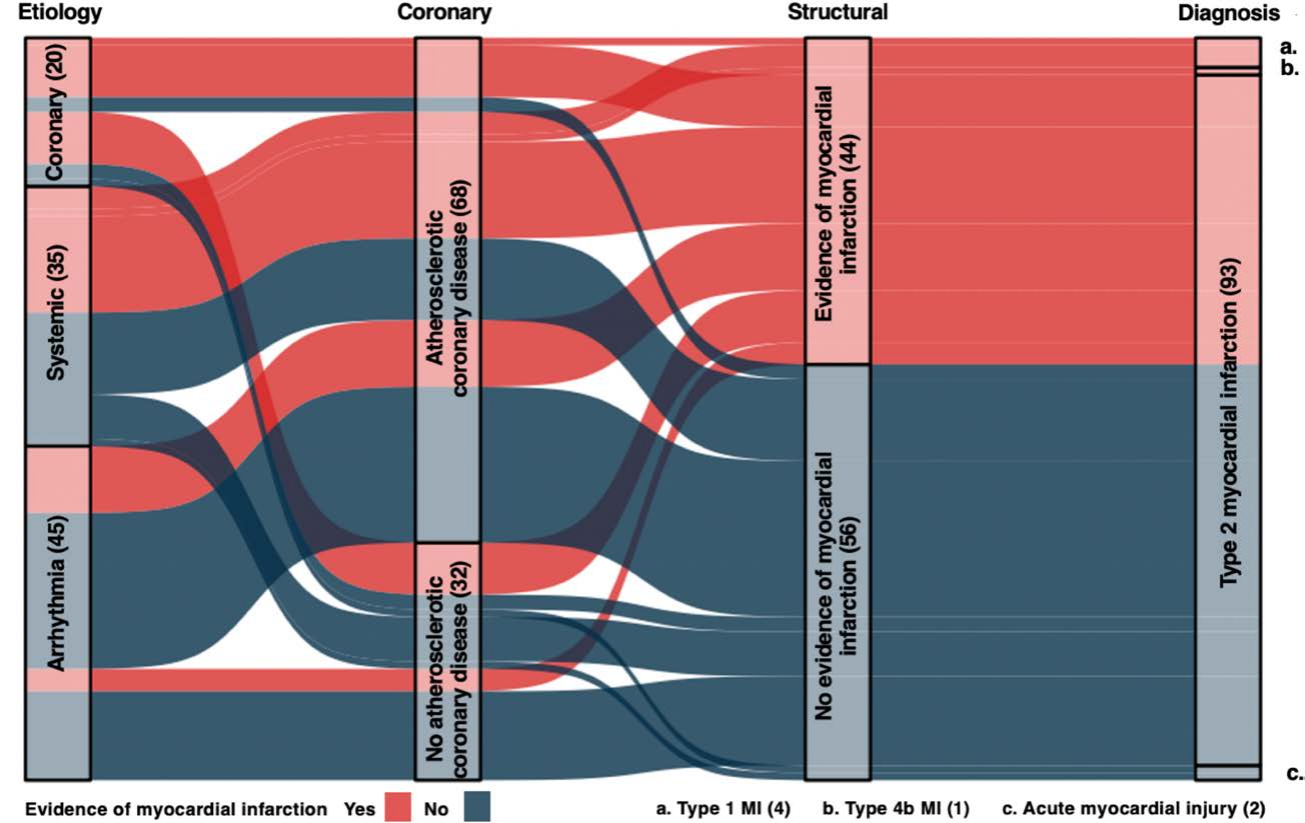
**Imaging findings and diagnostic reclassification in patients with a clinical diagnosis of type 2 myocardial infarction.** Alluvial plot illustrating the cause of supply-demand imbalance, presence of coronary disease on coronary imaging, and final adjudicated diagnosis according to the Fourth Universal Definition of Myocardial Infarction stratified according to evidence of myocardial infarction (MI) on cardiac imaging. The cause of supply-demand imbalance in type 2 myocardial infarction is categorized in 3 clinically relevant groups: coronary subgroup encompassing coronary artery dissection, coronary embolism, and vasospasm; systemic subgroup encompassing patients presenting for anemia, hypotension, severe hypertension, or hypoxemia; and arrhythmia subgroup encompassing supply-demand imbalance attributable to sustained bradyarrhythmia or tachyarrhythmia. MI indicates myocardial infarction.

Invasive coronary angiography and cardiac MRI were performed in 61% (61 of 100) and 78% (78 of 100), respectively, with the remainder undergoing CT angiography and echocardiography. The adjudicated diagnosis was type 2 myocardial infarction in 93 patients, who made up the study population. Seven patients were reclassified: 4 with type 1 myocardial infarction (attributable to atherosclerotic plaque rupture or thrombosis), 1 with type 4b myocardial infarction (caused by stent thrombosis), and 2 with acute nonischemic myocardial injury (1 attributable to myocarditis and 1 to takotsubo cardiomyopathy; Figure 2).

### Baseline Characteristics

In patients with type 2 myocardial infarction, symptoms of myocardial ischemia were present in 85% (79 of 93), with signs of myocardial ischemia on the 12-lead ECG in 76% (71 of 93; Table 1). Comorbidities were common, including hypertension (43% [40 of 93]) and atrial fibrillation (17% [16 of 93]). Coronary artery disease was previously recognized in 28% of patients (26 of 93), of whom 54% (14 of 26) had a previous myocardial infarction (Table 1). Median cardiac troponin I concentrations were 195 ng/L (62–760 ng/L) at presentation and 1165 ng/L (277–3782 ng/L) on repeat testing (Table 1). The commonest cause of supply-demand imbalance was arrhythmia (48% [45 of 93]), followed by systemic (32% [30 of 93]) and coronary (19% [18 of 93]) causes (Table S1). Clinical characteristics were comparable to those of a population of consecutive patients with an adjudicated diagnosis of type 2 myocardial infarction (Table S2).^15^

### Coronary Imaging

**Figure 3.**
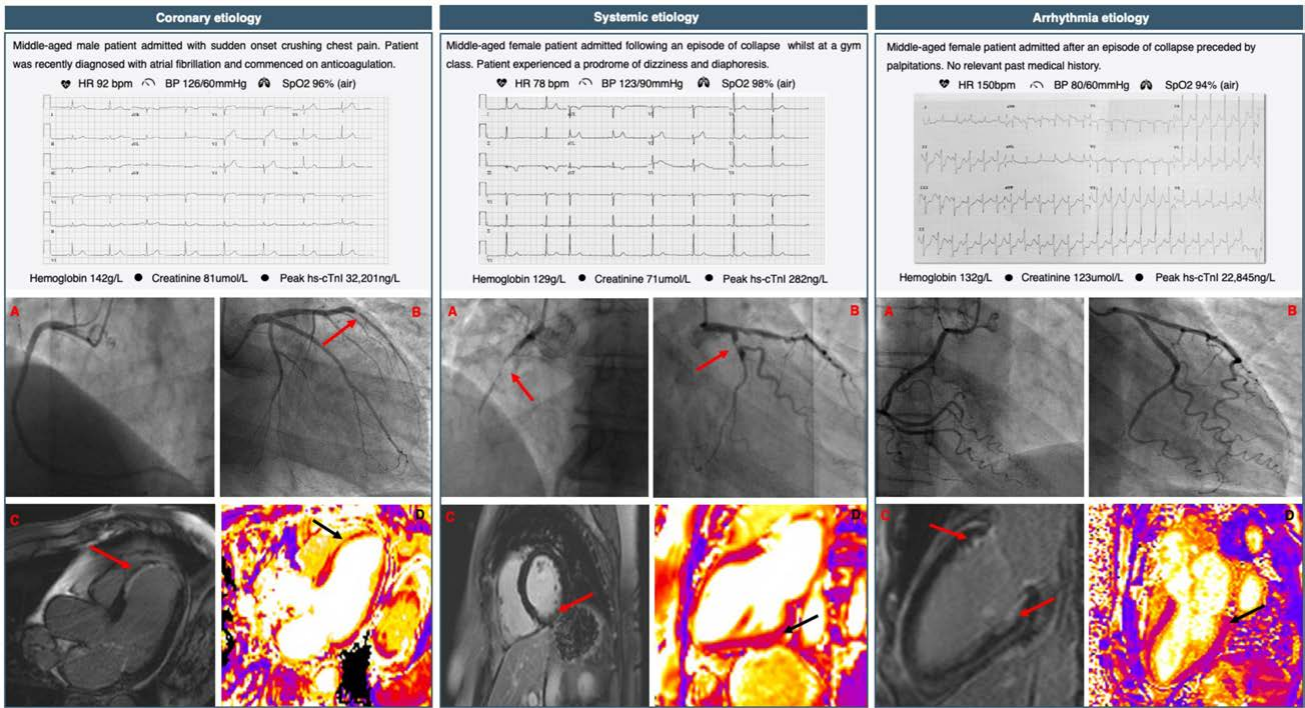
**Exemplar cases and imaging studies according to cause of type 2 myocardial infarction.** Patients with 3 differing causes of type 2 myocardial infarction showing clinical presentation data; study imaging, including invasive coronary angiogram; and cardiac magnetic resonance images. Coronary cause case: Invasive coronary angiogram (**A**) with evidence of coronary embolus with occlusion of the mid left anterior descending coronary artery (**B**). Cardiac magnetic resonance showed normal ventricular size and moderate impairment in left ventricular function (ejection fraction, 48%) with evidence of near-transmural late gadolinium enhancement in the anteroseptum (**C**). T2 value at the site of late gadolinium enhancement was elevated at 75.6 milliseconds, indicating an acute infarct (**D**). Systemic cause case: Invasive coronary angiogram showed evidence of 3-vessel obstructive coronary artery disease (**A** and **B**). Cardiac magnetic resonance showed normal ventricular size and function (ejection fraction, 64%) with evidence of subendocardial late gadolinium enhancement in the inferior wall (**C**). T2 value at the site of late gadolinium enhancement was elevated at 49.6 milliseconds, indicating an acute infarct (**D**). Arrhythmia cause case: Normal invasive coronary angiogram (**A** and **B**). Cardiac magnetic resonance showed normal ventricular size and function (ejection fraction, 62%) with evidence of basal subendocardial late gadolinium enhancement affecting the inferior, inferolateral, and anterior walls (**C**). T2 value at the site of late gadolinium enhancement was elevated at 51 milliseconds, indicating an acute infarct (**D**). BP indicates blood pressure; and HR, heart rate.

Participants had an invasive coronary angiogram (59%) or a CT coronary angiogram (41%) at a median of 3 days (1–11 days) from presentation. The prevalence of coronary artery disease was 68%, which was previously unrecognized in 60% of patients (Table 2). Patients with coronary artery disease were older (71 years [59–76 years] versus 56 years [50–67 years]; *P*<0.001) and more likely to be male (81% versus 78%; *P*<0.001) compared with those with no disease (Table 1). Obstructive coronary artery disease was present in 30%, with 6 patients found to have obstructive 3-vessel disease, 3 with left main stem disease, and 17 with proximal left anterior descending disease (Figure 3 and Table 2). Nonobstructive disease was present in 38%, with the remaining 32% having no evidence of coronary artery disease. There were no differences in baseline characteristics or prevalence of coronary disease between patients who had an invasive and those who had a coronary CT angiogram (Table S3).

**Table 2.**
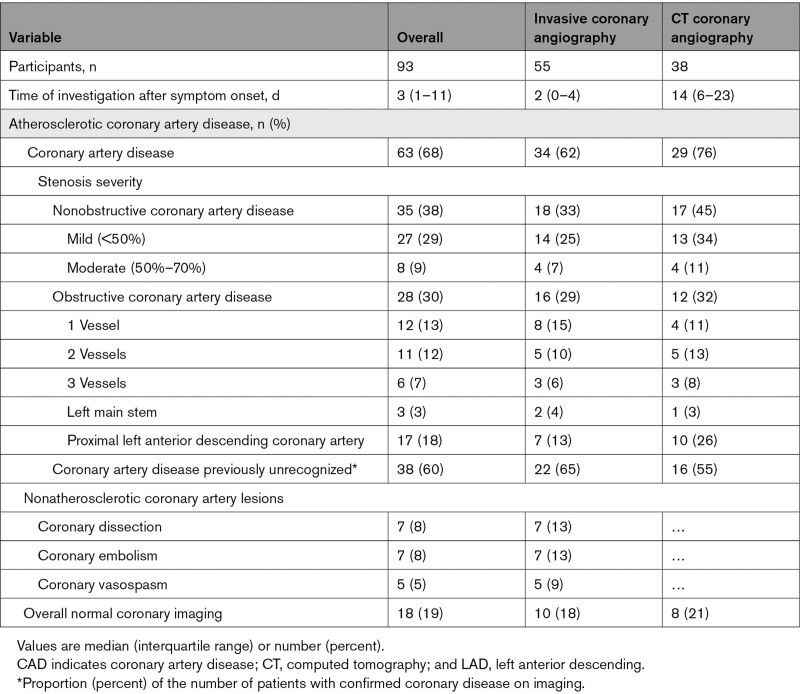
Findings on Coronary Imaging According to Study Investigation

Coronary mechanisms of type 2 myocardial infarction were identified in 19% of patients, with coronary embolism occurring in 8%, spontaneous coronary artery dissection in 7%, and coronary vasospasm in 5%. Overall, 19% (18 of 93) of patients had normal coronary imaging with no atherosclerosis or other coronary abnormalities. Coronary fractional flow reserve (9 of 55) and optical coherence tomography (7 of 55) were performed in some patients, with plaque rupture identified in 1 lesion (Table S4 and Figure S1).

### Cardiac Imaging

Participants had a cardiac magnetic resonance scan (77%) or echocardiogram (23%) at a median of 6 days (3–21 days) from presentation. Gadolinium-enhanced images were available in 68 patients, of whom 54% had evidence of late enhancement (Table 3). In the majority, the pattern was in keeping with myocardial infarction, and the remaining 4 patients had evidence of nonischemic enhancement (Figure 3 and Figure S2). We observed evidence of transmural and multiterritory myocardial infarction in some participants (Table S5 and Figure S2). In the 33 patients in whom T2 mapping was available, the median T2 value at the site of late gadolinium enhancement was 48.9 milliseconds (43.9–54.2 milliseconds) and was consistent with acute myocardial infarction in 58% (Table 3). With either imaging modality, a regional wall motion abnormality and evidence of myocardial infarction were observed in 30% and 42% of patients, respectively (Figure 2, Table 3, and Table S6).

**Table 3.**
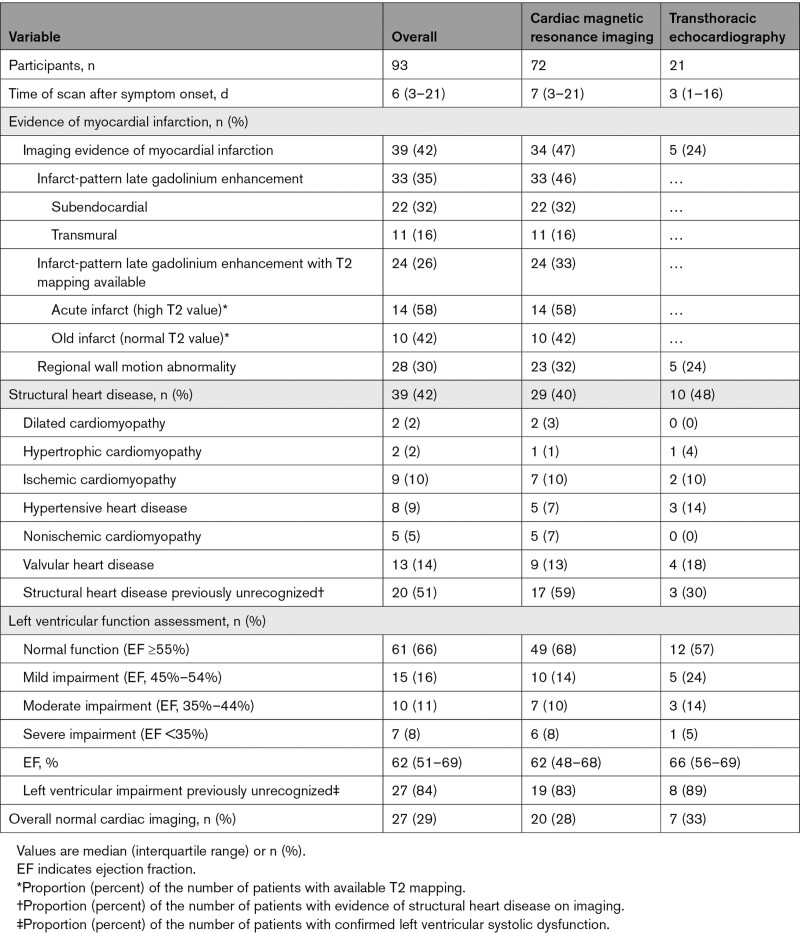
Findings on Cardiac Imaging According to Study Investigation

Left ventricular systolic dysfunction was present in 34% and was previously unrecognized in 84% of patients (Table 3). This was classified as mild in 16%, moderate in 11%, and severe in 7% of patients. Structural heart disease was observed in 42%, which was previously unrecognized in 51% of patients. Valvular heart disease was present in 14%, with ischemic cardiomyopathy and hypertensive heart disease identified in 10% and 9% of patients, respectively. Dilated, hypertrophic, and other forms of cardiomyopathy were observed less frequently (Table 3 and Figure S3). Overall, 29% of patients had normal cardiac imaging with no evidence of left ventricular dysfunction, structural heart disease, or myocardial infarction.

### Implications for Management

In patients with type 2 myocardial infarction, 19% (18 of 93) had normal coronary imaging, and 30% (28 of 93) had normal cardiac MRI or echocardiography. Both coronary and cardiac investigations were normal in 10 patients. These patients were more frequently female, were younger, and had fewer comorbidities (Tables S7 and S86).

**Figure 4.**
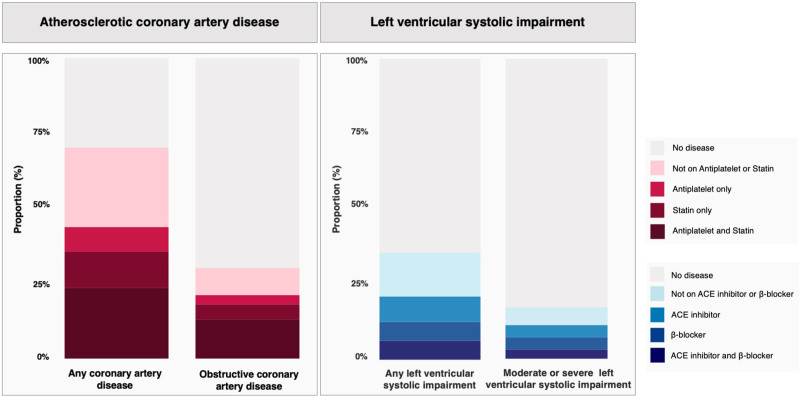
**Prior treatment in patients with coronary artery disease and left ventricular systolic impairment identified on coronary and cardiac imaging.** Proportion of patients with coronary artery disease or left ventricular impairment identified on coronary and cardiac imaging, respectively, stratified by prior treatment with evidence-based medical therapy. ACE indicates angiotensin-converting enzyme.

Only 33% (21 of 63) of patients with evidence of coronary artery disease and 40% (12 of 30) of patients with obstructive coronary artery disease were on prior antiplatelet and lipid-lowering therapies (Figure 4). In patients with left ventricular impairment, just 19% (6 of 32) were on angiotensin-converting enzyme inhibitor or angiotensin receptor blocker and β-blocker therapies at presentation, with only 3 of 17 patients with moderate or severe left ventricular impairment on these treatments.

Study imaging led to a change in management in 40% (29 of 72) of patients with coronary artery disease or left ventricular impairment. Of the 63 patients with coronary artery disease, 40% (25 of 63) of patients received new preventive therapy, and in the 32 patients with left ventricular impairment, 34% (11 of 32) had new therapy started (Figure S4). Anticoagulation and rate or rhythm control medications were started in 24 patients. All management was at the discretion of the attending clinician, with percutaneous coronary intervention (n=4), coronary artery bypass graft surgery (n=1), aortic valve replacement surgery (n=2), device implantation (n=3), and electrophysiology studies with ablation (n=2) undertaken in some patients.

## Discussion

We systematically performed coronary and cardiac imaging in patients diagnosed with type 2 myocardial infarction to determine the prevalence of coronary artery and cardiac disease. After imaging, the clinical diagnosis was reclassified to acute myocardial injury or type 1 or 4b myocardial infarction in 7 of 100 patients, with immediate implications for their management and treatment. In those with confirmed type 2 myocardial infarction, two-thirds had coronary artery disease and one-third had left ventricular impairment. In the majority, these abnormalities were previously unrecognized, with fewer than half prescribed evidence-based treatments. Although regional wall motion abnormalities or myocardial scar consistent with infarction was observed in nearly half of those with type 2 myocardial infarction, just 1 in 10 patients had both normal coronary and cardiac imaging. Taken together, these findings demonstrate the value of coronary and cardiac imaging and the substantial burden of coronary artery and cardiac disease in patients with type 2 myocardial infarction. This has major implications for the management and potential outcomes of this often underinvestigated and undertreated group of patients.

The diagnosis of type 2 myocardial infarction was introduced in 2007, partly in recognition that cardiac troponin concentrations were often raised in patients without evidence of coronary atherothrombosis.^1^ It is well recognized that patients with type 2 myocardial infarction have poor outcomes,^4,21–23^ with just one-third alive 5 years after the diagnosis.^24^ Although having a higher proportion of noncardiovascular deaths, patients with type 2 myocardial infarction are also at risk of future cardiovascular events at a level similar to that for patients with type 1 myocardial infarction.^23–25^ Furthermore, patients with type 2 myocardial infarction and known coronary artery disease have more cardiovascular events than patients with type 1 myocardial infarction.^7,8,23,25^ Despite these observations, the utility of the diagnosis of type 2 myocardial infarction has been questioned, with many clinicians uncertain about how to proceed with these patients and some dismissing the elevation of cardiac troponin as an unhelpful anomaly. Consequently, most patients with type 2 myocardial infarction do not routinely undergo cardiac imaging or receive additional cardiovascular therapies.^4,10–12,26^ To the best of our knowledge, this is the first prospective study in type 2 myocardial infarction to systematically evaluate and image the coronary arteries and the heart in all affected patients. Our data demonstrate a high prevalence of unrecognized and untreated coronary artery and cardiac disease, which likely has important prognostic implications.

We recruited a representative population of patients with type 2 myocardial infarction by screening consecutive patients with elevated cardiac troponin concentrations. However, we did not recruit many patients with concomitant frailty, advanced renal or hepatic impairment, or illness severity. Our enrollment criteria were designed to minimize participant risk but led to an unavoidable degree of case selection bias, and the prevalence of coronary artery and cardiac disease may have been underestimated. Despite this, our population had baseline characteristics comparable to those of individuals enrolled in an unselected consecutive patient population and are likely to reflect those patients who can undergo cardiac imaging in practice or who would be eligible for randomized trials of interventions in type 2 myocardial infarction.^15^

In more than half of patients with a diagnosis of type 2 myocardial infarction, we found no imaging evidence of any functional consequences of myocardial infarction such as a regional wall motion abnormality or scar formation. Although the sensitivity of cardiac imaging can be limited,^27^ the median cardiac troponin concentration in our population was >1000 ng/L. These observations bring into question whether it is appropriate or informative to diagnose type 2 myocardial infarction in all patients in whom myocardial ischemia and injury arise in the context of another condition. For example, in a patient with tachyarrhythmia who has neither coronary artery disease nor any functional consequences of acute ischemic myocardial injury, is the diagnosis of myocardial infarction helpful? If cardiac imaging does not identify coronary artery disease or infarction, then it is unlikely that the patient will benefit from therapies targeting coronary atherosclerosis. In this setting, the value of a diagnosis of type 2 myocardial infarction is questionable. This is a particularly important issue for patients in whom the label of myocardial infarction has major consequences for well-being, employment, and insurance, but there may be no immediate or long-term implications for treatment. In the setting of cardiac surgery, elevations in cardiac troponin are a universal finding and congruent with the insult of heart bypass and surgery. Consequently, imaging evidence of coronary artery disease and infarction is required to confirm the diagnosis of type 5 myocardial infarction,^4^ and one could argue that a similar approach is urgently needed in type 2 myocardial infarction in which the diagnosis is at least as challenging with similar implications for patients.

In clinical practice,^8,12^ fewer than one-third of patients with type 2 myocardial infarction are managed by cardiologists, and these patients are consistently less likely than those with type 1 myocardial infarction to undergo coronary or cardiac imaging.^4,8–10,28,29^ This is despite the fact that type 2 myocardial infarction is more challenging to diagnose, has a wide spectrum of underlying causes, and has more varied and less certain consequences. Our observations suggest that cardiac imaging can sometimes reclassify myocardial infarction and more often than not identify unrecognized coronary artery disease or left ventricular impairment. Given that there are established evidence-based treatments to prevent coronary events and heart failure, routine cardiac imaging in type 2 myocardial infarction could have major implications for treatment, with substantial potential downstream benefits for these patients.

Until randomized controlled trials comparing investigational strategies in type 2 myocardial infarction are undertaken, we would advocate that clinicians exercise pragmatism. According to our data, invasive or CT coronary angiography should be considered to identify prognostically important disease and to guide preventive therapies. Echocardiography should be considered in all patients, with cardiac magnetic resonance reserved for those in whom the diagnosis remains unclear. In patients with few or no cardiovascular risk factors and marked physiological stress such as tachyarrhythmia or in whom prognosis is poor because of the primary illness, comorbidity, or frailty, it may be reasonable to defer investigation altogether.

Our study has important limitations. As a result of contraindications, patient choice, and public health restrictions, we were unable to perform cardiac magnetic resonance in all patients. Echocardiography was performed in the remainder of patients, and we acknowledge that the use of regional wall motion abnormality as a surrogate for myocardial infarction may lead to underdiagnosis. Although selection bias is likely, participant screening was systematic, and the population recruited is likely to be representative of those who would be considered for cardiac imaging in clinical practice. Invasive intracoronary imaging was at the discretion of the attending cardiologist, and we were able to undertake this in only a limited number of patients. It is therefore possible that we may have missed some patients who had atherothrombotic events and type 1 myocardial infarction.

## Conclusions

Systematic imaging in patients with type 2 myocardial infarction identified coronary artery disease in two-thirds and left ventricular systolic dysfunction in one-third. This substantial burden of unrecognized disease underlines the importance of comprehensive assessment in patients with type 2 myocardial infarction, with the prospect of disease reclassification and the identification of opportunities for evidence-based preventive treatments.

## Article Information

### Acknowledgments

The authors are grateful to the staff of the Edinburgh Heart Center, Cardiac Catheter Laboratories, the BHF Cardiovascular Biomarker Laboratory, and the Edinburgh Imaging Facility for their support in delivering the DEMAND-MI study. They also extend thanks to the Edinburgh Imaging Academy team led by Professor Andrew Farrall who helped to develop the DEMAND-MI online educational resource.

### Sources of Funding

The DEMAND-MI study was funded by the British Heart Foundation (FS/16/75/32533). Drs Bularga and Wereski are supported by Clinical Research Training Fellowships (MR/V007254/1 and MR/V007017/1, respectively) from the Medical Research Council. Dr Taggart is supported by a Research Excellence Award from the British Heart Foundation (RE/18/5/34216). Dr Williams is supported by the British Heart Foundation (FS/ICRF/20/26002). Dr Newby is supported by the British Heart Foundation (CH/09/002, RG/16/10/32375, RE/18/5/34216) and is the recipient of a Wellcome Trust Senior Investigator Award (WT103782AIA). Dr Dweck is the recipient of the Sir Jules Thorn Award for Biomedical Science (15/JTA). Dr Mills is supported by a Chair Award (CH/F/21/90010), Program Grant (RG/20/10/34966), and a Research Excellent Award (RE/18/5/34216) from the British Heart Foundation. Dr Chapman receives support from a Starter Grant for Clinical Lecturers by the Academy of Medical Sciences (SGL021/1075).

### Disclosures

Dr Mills reports research grants awarded to the University of Edinburgh from Abbott Diagnostics and Siemens Healthineers outside the submitted work, as well as honoraria from Abbott Diagnostics, Siemens Healthineers, Roche Diagnostics, and LumiraDx. Dr Williams has received speaker fees from Cannon Medical Systems. All other authors report no conflicts.

### Supplemental Material

Supplemental Methods

Tables S1–S8

Figures S1–S4

Appendix: DEMAND-MI Study Protocol

References 30–39

## Supplementary Material
