# Supplementary material for: Coronary Artery and Cardiac Disease in Patients With Type 2 Myocardial Infarction: A Prospective Cohort Study
Source: Circulation. 2022 Mar 28;145(16):1188–200. doi: 10.1161/CIRCULATIONAHA.121.058542 (PMC9010024; doi:10.1161/CIRCULATIONAHA.121.058542)
Supplement: Supplementary file 1 [file cir-145-1188-s001.pdf]

SUPPLEMENTAL MATERIAL

Coronary artery and cardiac disease in patients with type 2 myocardial infarction

A prospective cohort study

Coronary disease and type 2 myocardial infarction

Anda Bularga, MD,[1*] John Hung, MD,[1*] Marwa Daghem, MD,[1] Stacey Stewart, MSc,[1] Caelan Taggart, MD,[1] Ryan Wereski, MD,[1] Trisha Singh, MD,[1] Mohammed N Meah, MD,[1] Takeshi Fujisawa, PhD,[1] Amy V Ferry, PhD,[1] Justin Chiong, MD,[1] William S Jenkins, MD,[1] Fiona E Strachan, PhD,[1] Scott Semple, PhD,[2] Edwin J R van Beek, PhD,[2] Michelle Williams, MD,[1,2] Damini Dey PhD,[3] Chris Tuck BSc,[1] Andrew H Baker PhD,[1] David E Newby, MD,[1] Marc R Dweck, MD,[1] Nicholas L Mills, MD,[1,4] Andrew R Chapman, MD[1]

[1] *BHF Centre for Cardiovascular Science, University of Edinburgh, United Kingdom*
[2] *Edinburgh Imaging, University of Edinburgh, United Kingdom*
[3] *Cedars-Sinai Medical Center, Biomedical Imaging Research Institute, Los Angeles, United States of America*
[4] *Usher Institute, University of Edinburgh, United Kingdom*

*These authors contributed equally

[1] *BHF Centre for Cardiovascular Science, University of Edinburgh, United Kingdom*
[2] *Edinburgh Imaging, University of Edinburgh, United Kingdom*
[3] *Biomedical Imaging Research Institute, Cedars-Sinai Medical Center, Los Angeles, United States of America*
[4] *Usher Institute, University of Edinburgh, United Kingdom*

Correspondence and requests for reprints:
Dr Andrew R Chapman
BHF Centre for Cardiovascular Science
Chancellors Building
Royal Infirmary of Edinburgh
Edinburgh EH16 4SA
United Kingdom

Tel: +44-131-242-6515
Fax: +44-131-242-6379
Email: a.r.chapman@ed.ac.uk
Twitter: @chapdoc1

DEMAND-MI Educational resource: *www.ed.ac.uk/Edinburgh-imaging/demand-mi-cases*

Contents

Supplemental Methods Pages 3-13

Supplemental Tables

Table S1 Page 14

Table S2 Page 15

Table S3 Page 16

Table S4 Page 17

Table S5 Page 18

Table S6 Page 19

Table S7 Page 20

Table S8 Page 22

Supplemental Figures

Figure S1 Page 23

Figure S2 Page 24

Figure S3 Page 25

Figure S4 Page 26

Appendix – DEMAND-MI study protocol Pages 27-53

Supplemental Methods

1. Study Design

Design: Prospective cohort study **Setting:** Royal Infirmary of Edinburgh

Study population: Consecutive patients with acute myocardial injury (defined as a rise and or fall in cardiac troponin concentration on serial testing, with at least one value >99th centile) and symptoms or signs of myocardial ischemia on the electrocardiogram where the likely mechanism of injury was thought to be myocardial oxygen supply and demand imbalance (e.g secondary to hypoxemia, hypotension, tachyarrhythmia or anemia).[4] Signs of myocardial ischemia were defined as new electrocardiogram ST segment (elevation or depression) or T wave (inversion) changes in-keeping with acute ischemia as per pre-defined criteria.[4] Patients were identified through screening of cardiac troponin measurements using the electronic patient record and laboratory databases at the recruiting site. All screened patients were recorded in a screening log. Patients who met both the inclusion and exclusion criteria, were approached and those who provided consent comprised the study population and were allocated a unique study number.

Inclusion criteria:

- Unscheduled hospital admission with acute myocardial injury (defined as a rise and or fall in high-sensitivity cardiac troponin I concentrations on blood testing)
- A suspected etiology of myocardial oxygen supply and demand imbalance with symptoms or signs of myocardial ischemia

Exclusion criteria:

- Unable or unwilling to give informed consent
- Women who are pregnant or breastfeeding will not be enrolled into the trial.

- Probable type 1 myocardial infarction
- Renal impairment (estimated glomerular filtration rate ≤30ml/min/1.73m^2)
- Severe hepatic impairment
- Frailty with inability to self-transfer (determined using Katz Index)

2. Imaging Protocols

2.1 Coronary imaging

Invasive coronary angiography was performed via the radial or femoral artery. In patients with one or more stenoses in a major epicardial vessel, a coronary pressure guidewire (PressureWire™ Aeris™, St. Jude Medical, St. Paul, Minnesota) was undertaken where feasible to determine distal coronary pressure and the fractional flow reserve (FFR) calculated at maximal adenosine-induced (intravenous 140 μg/kg/min) hyperaemia.[29] Optical coherence tomography (OCT) was performed where possible with pullback at 20 mm/s to identify features consistent with vulnerable plaque or recent plaque rupture.[30]

Coronary computed tomography angiography was performed using a 128 multidetector row CT scanner (Siemens Biograph, Siemens Healthcare, Erlangen, Germany). Patients with a heart rate exceeding 65 beats/min were administered oral or intravenous beta-blockade and all received sublingual glyceryl trinitrate (300μg) immediately prior to electrocardiogram-gated coronary computed tomography angiography during breath-hold. A bolus of 80-100 mL of contrast (400 mg/mL; Iomeron, Bracco, Milan, Italy) was injected intravenously at 5 mL/s.

2.2 Cardiac imaging

Cardiac magnetic resonance imaging (MRI) was performed in all patients without contraindication using a 3T scanner (MAGNETOM Verio, Siemens AG, Healthcare Sector, Erlangen, Germany). The MRI scan consisted of localisers, axial and coronal HASTE images, and standard breath-held and ECG-gated cine sequences. These were acquired with standard steady-state free precession sequences in long- and short-axis orientations as described previously.[31] The late gadolinium enhancement and T2-weighted (MyoMaps) imaging techniques were used to identify regions of new or old myocardial infarction as well as other patterns of injury. T2 measurements were taken from septal segments and from the site of late

gadolinium enhancement where this was present. Reference T2 values were obtained from a cohort of ten healthy volunteers who underwent cardiac MRI on the same 3T scanner (mean T2 value 38.7±3 ms).[32] Where feasible and coronary anatomy was known with no contraindications present, patients underwent stress perfusion imaging following administration of a 0.2 mmol/kg Gadolinium contrast bolus and 0.4mg (5ml) of peripheral Regadenoson (Rapiscan™) 8-15 minutes post contrast administration. Late gadolinium enhancement images were acquired in 2 chamber and 4 chamber short and long-axis views and in two phase encoding directions. T2 mapping (MyoMaps) was acquired in the 2 chamber and 4 chamber long-axis orientation. The late gadolinium enhancement and T2-weighted imaging techniques were used to identify regions of new or old myocardial infarction as well as other patterns of injury.

Figure A (Supplemental Methods) showing the three major components to the cardiac magnetic resonance imaging protocol:

**Major components**	**Structural imaging**	**T1-weighted coronary imaging and T2 mapping**	**Late gadolinium enhancement**
Pulse sequence	Cine MRI	Pre contrast imaging	Fast IR-prepared gradient echo post contrast
Typical images	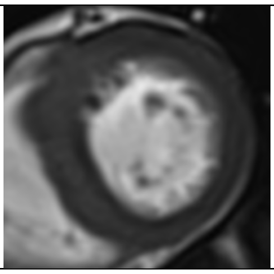	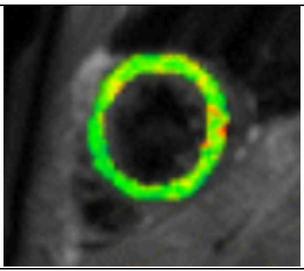	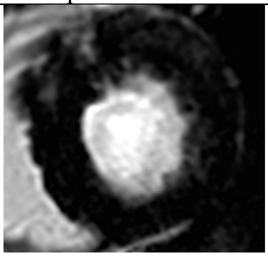
Rationale	Quantify LV size, mass and systolic function	To assess for coronary thrombus and myocardial oedema (above)	Identify the presence of mid-wall late gadolinium enhancement as a marker of LV decompensation

The imaging protocol consisted of:

1. Localiser sequences
2. Left ventricular imaging (4 chambers, 2 chambers and 3 chambers long axis cines)
3. Interleaved angiogram and T1-weighted coronary imaging (CATCH)
4. T2 myocardial mapping (2, 3 and 4 chamber long axis images)
5. Stress perfusion imaging (with *Regadenoson*)
6. Gadolinium contrast administration
7. LV (left ventricular) short axis cine stack
8. Late gadolinium enhancement imaging

Table A (Supplemental Methods) summarizing the study cardiac MRI sequences:

	Number of acquisitions	Estimated time	Time post-contrast
Position subject within scanner, ensure high quality ECG signal and IV cannula patency		5 mins	
Sequence			
Localiser	1		
Site specific method of localisers to derive appropriate cardiac planes	1-5	1-3 mins	
4 chamber (horizontal long axis) cine	1	2 min	
2 chamber (vertical long axis) cine	1	2 min	
3 chamber cine	1	2 min	
Interleaved angiogram and T1-weighted coronary imaging (CATCH sequence)	1	12 mins	
T2 myocardial mapping (2, 3 and 4 chamber long axis images)	3	3 mins	
Inject regadenoson			
Inject contrast (0.15mmol/kg)			
Stress perfusion imaging (short axis images, basal mid and apex)		3 mins	1-3
LV short axis stack cine – 8mm slice thickness, no gap	10	4 mins	3-7
TI scout – to determine optimal TI	1	1 min	7-8
GRE-T1 LGE LV short axis stack – 8mm slice thickness, no gap	14-15	7 mins	8-15
Phase swap GRE-T1 LGE LV short axis stack*	14-15	7 mins	15-22
Long axis GRE-T1 LGE images	3	3 mins	22-25
Total scan time			
Standard protocol		55 mins	

3. Image Analysis

3.1 Coronary imaging

Coronary computed tomography angiography (CCTA) imaging was reviewed on a dedicated post processing workstation (Vitrea Advanced, v6.9.68.1, Vital Images, US) by experienced observers (MCW, EJRB). Reconstructions of contrast-enhanced images were performed on the best phase in mid-diastole or end-systole based on established techniques.[18] The CCTA dataset was anonymized and exported in a Digital Imaging and COmmunications in Medicine (DICOM) format to allow quantitative measurement of plaque subtypes. Plaque analysis was performed using semi-automated software (Autoplaque version 2.5, Cedars-Sinai Medical Center, Los Angeles, USA) by a trained observer (MNM). This method has excellent observer agreement, even in patients with advanced coronary disease, and has been validated against intravascular ultrasound.[33,34,35] Coronary artery center lines were extracted in a semi-automated fashion for each major artery and any tributary of >2 mm diameter with visually observed disease. A region of interest was placed in the aorta to define blood pool attenuation. Coronary artery segments were defined manually according to the Society of Cardiovascular Computed Tomography guidance, using side-branches to mark progression from proximal to mid and distal segments.[36,37] Segments with visible disease were manually identified, and vessel wall and plaque constituents were automatically determined using scan-specific thresholds with manual adjustments made as required. In an exploratory analysis where CT scans were of satisfactory diagnostic quality, an assessment of the functional consequences of coronary artery stenosis was made using the fractional flow reserve for coronary computed tomography angiography (FFR-CT) technique, using the HeartFlow™ platform.[38]

3.2 Cardiac imaging

Cardiac magnetic resonance studies were analysed offline using Circle CVI (Circle Cardiovascular Imaging, CVI42 v5.3.6, Calgary Canada). T2 maps, and cine-derived volumetric and functional sequences was analysed by experienced observers (MRD, WJ, ARC, JH, MD, TS, AB). Endocardial and epicardial borders were manually defined on all the conventional short-axis images for volumetric and wall motion measurements and were then copied to corresponding LGE sequences for analysis with minimal manual adjustments.[31] Regions of interest (ROIs) were determined using the standard 16-segment cardiac model with septal native T2 values. For patients with previous myocardial infarction or a new diagnosis of infarction, the region of interest (ROIs) was defined in the remote myocardium. We determined left ventricular ejection fraction using standard volumetric analysis of a short axis stack sequence. A blinded analysis of cardiac MRI was undertaken by an independent expert (WJ), to validate the assessment of late gadolinium enhancement pattern. The blinded analysis involved assessment of late gadolinium enhancement presence and pattern (ischaemic or non-ischaemic [mid-wall or epicardial]).

Echocardiography was undertaken in line with guidance from the British Society of Echocardiography.[19] Standard image acquisition including basal dimensions, an assessment of ventricular function with estimated left ventricular ejection fraction using Simpson's biplane, and assessment of valvular dysfunction using standard doppler imaging was obtained in line with requirements for a standard examination.

4. Diagnostic Adjudication

In all patients with type 2 myocardial infarction the primary aetiology of supply-demand imbalance was classified using the following categories of reduced myocardial perfusion: coronary artery dissection, coronary embolism, coronary vasospasm, sustained

bradyarrhythmia, hypotension or shock, hypoxemia and severe anemia (anemia), or increased myocardial oxygen demand: severe hypertension with or without left ventricular hypertrophy (severe hypertension) and sustained tachyarrhythmia (tachyarrhythmia).[4] When defining the primary causes of supply-demand imbalance abnormal levels of relevant clinical parameters were considered, however, strict cut offs for supply-demand clinical variables, such as blood pressure, oxygen saturations or hemoglobin concentrations, were not used as the level at which these lead to demand ischemia will be patient specific. The etiology of supply-demand imbalance in type 2 myocardial infarction was categorized in three clinically relevant subgroups: coronary, systemic and arrhythmia etiologies. Patients with type 2 myocardial infarction due to coronary artery dissection, embolism or vasospasm were grouped in a '*coronary etiology'* category. Patients with myocardial infarction in response to an acute systemic illness, presenting with anemia, hypotension, hypoxemia, or severe hypertension, were grouped together in a *'systemic etiology'* category. Patients with myocardial infarction due to primary brady- or tachyarrhythmia were included in a *'arrhythmia etiology'* category.

5. Data protection impact assessment for the DEMAND-MI study cases

THE UNIVERSITY *of* EDINBURGH

Assessment Details

Name R - DEMAND-MI: online data supplement

Respondent Christopher Tuck

Date Completed 07/05/2021 12:59

Approver Rena Gertz

Stage Completed

Result Approved

Assessment Questions

1 Overview

1.1 **Research Outline**
Summarise what the research aims to achieve and what type of processing it involves. You may find it helpful to refer or link to other documents, such as a project proposal. Explain why and how the processing of information is necessary to that work?

Response
DEMAND-MI is a clinical trial looking to see if patients who have been diagnosed as having a type 2 heart attack also have coronary artery disease. For publication and research purposes, it is planned that case studies (summary data and anonymised imaging) is made available for review.

To achieve this, personal data will be processed to deidentify the records.

1.2 **Describe the information flow**
You should describe the collection, use and deletion of personal data here and it may also be useful to refer to a flow diagram or another way of explaining data flows – where you are getting the data from, where it will be stored and where it could be transferred to. You should also say how many individuals are likely to be affected by the project.

e.g. Data will be collected from research participants via online forms

↓

Data will be stored encrypted on departmental drives

↓

Pseudonymised dataset will be provided to Department X along with report

Response
Data from DEMAND-MI will be summarised for key variables
↓
Imaging data will be added and combined into a case report
↓
Deidentified case reports will be made available on CVS imaging pages

2 Compliance with Privacy Laws

2.1 **Check**
Data Protection legislation is relevant to any DPIA, and this section forms the data protection compliance check which should always be carried out. The Data Protection Officer will be able to advise you on the relevance of other privacy laws.

2.2 **What type of personal data are you processing?**
For guidance on what personal data is, consult the ***definitions.***

Response
Patient data

Justification
None

2.3 **Common Law duty of confidence**
This only applies when you use, for example medical data or similar types of data that individuals would not expect to see disclosed. Describe how you have obtained consent.

Response
Yes

Justification
Participants are consented into the trial using a Participant Information Sheet and Consent Form

2.4 **Human Tissue Act and the Medicines for Human Use (Clinical Trials) Regulations**
Note: This only applies to medical research. Describe how you have obtained consent.

Response
No

Justification
None

2.5 **List the personal data you are going to process?**
For guidance on what personal data is, consult the ***definitions.***

Response
Please see attached a draft of the anonymisation process that will be carried out. This process has been sent to the sponsor for review who had no comment (also attached).

2.6 **Which of the legal bases in Article 6 (1) will provide a lawful basis for the processing?**
For research the legal basis will typically be task carried out in the public interest or 'public task'.

Consult the document ***Guidance – how to determine the legal basis for processing personal data*** *for information on the other potential legal bases.*

Response
Public Tasks

Justification
None

2.7 **Special Categories of Personal Data**
If special categories of personal data are going to be processed, which of the legal bases in Article 9 (in addition to the Article 6(1) legal bases) will provide a legitimate basis for that processing? Consult the ***special category guidance*** *for information on determining the legal basis for special category data.*

For research the legal basis for special category personal data will typically be Article 9 (2) (j) – necessary for research in the public interest or 'archive, statistical and research purposes'.

Note – special categories of personal data are personal data consisting of information as to (a) the racial or ethnic origin of the data subject, (b) political opinions, (c) religious beliefs, (d) Trade Union membership, (e) physical or mental health, (f) sexual life, (g) genetic data and (h) biometric information.

Response
Archive, statistical and research purposes

Justification
None

2.8 **How are individuals being made aware of how their personal data will be used?**
How are individuals being made aware of how their personal data will be used? If you supply participants with a Participant Information Sheet (PIS), please attach the PIS.

There is a template PIS available within the ***Research and the General Data Protection Regulation*** *guidance.*

Response
Participants are provided a PIS and consent form

2.9 **Does the activity involve the use of existing personal data for new purposes?**

Response
No

Justification
None

2.10 **Is there a way to check that the data collection procedures are adequate, relevant and not excessive in relation to the purpose for which the data will be processed?**

Response
Not Applicable

2.11 **How will the personal data be checked for accuracy?**

Response
Not applicable - this processing task is to remove personal data

2.12 **Will there be set retention periods in place in relation to the storage of the personal data?**

Will there be set retention periods in place in relation to the storage of the personal data? If 'Yes', you will need to include details of this in your PIS.

If you applying the research exemption that the data in intended for future use, you can select 'No'.

Response

No

Justification
None

2.13 **What technical and organisational security measures will be in place to prevent any unauthorised or unlawful processing of the personal data?**

Response
All personal data will be processed on secure university or NHS drives.

2.14 **Has the personal data been evaluated to determine whether its processing could cause unwarranted damage or distress to data subjects?**

Response
Yes

Justification
The personal data will be removed. Any remaining data will be clinically relevant and not include specific information that could cause distress or damage.

2.15 **Do you use a data processor?**

Response
No

Justification
None

2.17 **Will you share the data with an external third party?**

Response
Yes

Justification
Data will be made available as a supplement to the publication. Data will be de-identified so not considered personal.

2.18 **Do you have a data sharing agreement in place**
If you don't have a data processing agreement in place, please contact the relevant team below:

- *If the data processing relates to research (other than industry-related research), please contact the Edinburgh Research Office, at RSO.Contracts@ed.ac.uk*
- *If the data processing relates to the Roslin Institute, please contact the dedicated legal team the Roslin Institute at EBCampusLegal@ed.ac.uk*
- *For any other data processing activities (including industry-related research) please contact the Legal Services team at legalservices@ed.ac.uk*

*In each case, please use the subject '****DPIA – Request for Data Sharing Agreement'*** *in your email.*

Response
No - all data anonymous to third parties

2.19 **Will you be transferring personal data to a country outside of the European Union or the European Economic Area (EEA)?**
Countries in the EU
Countries in the EEA

Response
No

Justification
None

2.23 **If the data will be anonymised, is it likely that a 'motivated intruder' will be interested in attempting re-identification by linking the data with other information available to them?**
For guidance on 'motivated intruders', please see ***here.***

Response
No

Justification
This is possible - as by definition a motivated intruder is interested in re-identification but it is unlikely that there is a "motivated intruder" for this set of data.

2.24 **From the Data Protection compliance check in this section we have concluded:**
Have you satisfied all the requirements asked for above?

Response

We have concluded that the research project is data protection compliant.

Justification
None

3 Screening

3.1 **Screening**
Answer the following questions to determine any potential privacy risks in your research.

You will then be asked to explain what practical steps you will take to ensure that you identify and address privacy risks. Who should be consulted about potential risks (members of your research team, supervisor, research sponsor...)?

3.2 **Will the research involve the collection of new identifiable or potentially identifiable information about individuals?**

Response
Yes

Justification
Yes, the main research collected new personal data, however this DPIA focuses solely on the processing required to anonymise the data in preparation for training case reports.

3.3 **Will the research compel individuals to provide information about themselves, i.e. where they will have little awareness or choice?**

Response
No

Justification
None

3.4 **Will identifiable information about individuals be shared with other organisations or people who have not previously had routine access to the information?**

Response
No

Justification
None

3.5 **Are you using information about individuals for a purpose it is not currently used for or in a new way, i.e. using data collected to provide care for an evaluation of service development?**

Response
No

Justification
None

3.6 **Will there be new or significantly changed consolidation, inter-linking, cross-referencing or matching of personal data from multiple sources?**

Response
No

Justification
None

3.7 **Where information about individuals is being used, would this be likely to raise privacy concerns or expectations, i.e. will it include health records, criminal records or other information that people may consider to be sensitive and private and may cause them concern or distress?**

Response
No

Justification
None

3.8 **Will the project require you to contact individuals in ways which they may find intrusive, i.e. telephoning or emailing them without their prior consent?**

Response
No

Justification
None

3.9 **Will the project result in you making decisions in ways which can have a significant impact on individuals, i.e. will it affect the care a person receives?**

Response
No

Justification
None

3.10 **Does the project involve you using new technology which might be perceived as being privacy intrusive, i.e. using biometrics, facial recognition or automated decision making?**

Response
No

Justification
None

3.11 **Is a service being transferred to a new supplier (re-contracted) and the end of an existing contract?**

Response
No

Justification
None

3.12 **Is processing of identifiable/potentially identifiable data being moved to a new organisation (but with same staff and processes)?**

Response
No

Justification
None

3.13 **Consultation to address privacy risks**
Explain what practical steps you will take to ensure that you identify and address privacy risks highlighted in the preceding questions. Who should be consulted about potential risks (members of your research team, supervisor, research sponsor...)?

Response
The research team have had internal discussions to reduce privacy risks when creating case reports.

4 Risk identification

4.1 **Risk identification and assessment**
Together with the other stakeholders, now list all the risks you can identify. Below, you will find a list of common risks. If any of these apply, choose them by answering 'yes' and providing a short description of the risk and how you would mitigate the risk.

If the risk applies – select 'Yes'. As a result you will be require to:

1. Provide a brief explanation / consequence of the risk occurring
2. Provide a brief explanation of the mitigating factors that will be undertaken to either eliminate or lower the risk
3. In considering the defined mitigation measures, determine the likelihood of the risk occurring to be Low, Medium or High
4. In considering the defined mitigation measures, determine the impact on the data subjects and on the University to be Low, Medium or High

If the risk does not apply, select 'No'

4.2 **Possibility that information is shared inappropriately.**
If this risk applies, click 'yes' and give a brief explanation of why the risk applies.

If the risk does not apply, click 'no'

Response
Yes

Justification
If case reports are not suitably processed for anonymisation, information pertaining to an individual could be available publically

4.3 **What can you do to eliminate or at least reduce the risk?**
Explain all mitigation measures you will put in place.

Response
A minimal dataset will be provided. All direct identifiers will be removed and indirect identifiers removed if likely that, in combination, they would be identifiable. The draft process for anonymising data case reports is included in this DPIA.

4.4 **Is the overall residual risk after the mitigation measures low, medium or high? Provide an explanation for your choice.**

Response
Low

Justification
Established methods on anonymising datasets (https://trialsjournal.biomedcentral.com/articles/10.1186/s13063-017-2382-9) will be used. A minimal, standardised dataset will also be created which reduces risk,

---

Risks

Inherent Risk Level
2.0 - Low Impact / Low Probability

Residual Risk Level
2.0 - Low Impact / Low Probability

Stage
Identified

4.5 **Personal data may be used for a new and different purpose without the knowledge of the data subjects, perhaps due to a change in the context in which the data is used.**
If this risk applies, click 'yes' and give a brief explanation of why the risk applies.

If the risk does not apply, click 'no'

Response
No

Justification
None

4.8 **New surveillance methods such as CCTV, email monitoring etc. may be an unjustified intrusion on people's privacy.**
If this risk applies, click 'yes' and give a brief explanation of why the risk applies.

If the risk does not apply, click 'no'

Response
No

Justification
None

4.11 **Actions taken against individuals as a result of collecting information about them might be to their detrment or cause damage/distress.**
If this risk applies, click 'yes' and give a brief explanation of why the risk applies.

If the risk does not apply, click 'no'

Response
No

Justification
None

4.14 **People cannot participate in research anonymously because participants may become identifiable again due to data linkage, low participant numbers, geographical location, transfer of data, or access of data.**
If this risk applies, click 'yes' and give a brief explanation of why the risk applies.

If the risk does not apply, click 'no'

Response
Yes

Justification
Publication of any data into the public increases risk, even after anonymisation techniques are carried out. This risk is anticipated to be low.

4.15 **What can you do to eliminate or at least reduce the risk?**
Explain all mitigation measures you will put in place.

Response
Robust anonymisation and using the a minimal dataset ensures that it is unlikely to link the data to a natural person.

4.16 **Is the likelihood of the risk manifesting after the mitigation measures low, medium or high? Provide an explanation for your choice.**

Response
Low

Justification
Initial risk is low, plus it unlikely that any additional published data will significantly increase risk of reidentification.

Risks

Inherent Risk Level
2.0 - Low Impact / Low Probability

Residual Risk Level
2.0 - Low Impact / Low Probability

Stage
Identified

4.17 **Vulnerable people may be particularly concerned about the risks of identification or the disclosure of information if anonymity is what people were led to expect.**
If this risk applies, click 'yes' and give a brief explanation of why the risk applies.

If the risk does not apply, click 'no'

Response
No

Justification
None

4.20 **Collecting information, matching and linking identifiers or whole datasets might mean that data are no longer anonymous if anonymity is what people were led to expect.**
If this risk applies, click 'yes' and give a brief explanation of why the risk applies.

If the risk does not apply, click 'no'

Response
No

Justification
None

4.23 **Excess information collection or information not properly managed can lead to creation of duplicate records.**
If this risk applies, click 'yes' and give a brief explanation of why the risk applies.

If the risk does not apply, click 'no'

Response
No

Justification
None

4.26 **If a retention period is not established information might be used for longer than necessary.**
If this risk applies, click 'yes' and give a brief explanation of why the risk applies.

If the risk does not apply, click 'no'

Response
No

Justification
None

4.29 **The use of biometric information or potentially intrusive tracking technologies may cause increased concern and cause people to avoid engaging with the University.**
If this risk applies, click 'yes' and give a brief explanation of why the risk applies.

If the risk does not apply, click 'no'

Response
No

Justification
None

4.32 **Public distrust about how information is used can damage the University's reputation and lead to less willingness to participate.**
If this risk applies, click 'yes' and give a brief explanation of why the risk applies.

If the risk does not apply, click 'no'

Response
Yes

Justification
When processing personal data from research (even good practice such as anonymisation) it is vital to ensure that data is handled safely and securely. Any real or perceived misuse can be damaging to the reputation of the University and researchers.

4.33 **What can you do to eliminate or at least reduce the risk?**
Explain all mitigation measures you will put in place.

Response
It is important to ensure transparency about the process for anonymisation. Documenting the anonymisation process and also completion of the DPIA can create trust and uphold the reputation of the University and its researchers.

4.34 **Is the overall residual risk after the mitigation measures low, medium or high? Provide an explanation for your choice.**

Response
Low

Justification
None

Risks

Inherent Risk Level
2.0 - Low Impact / Low Probability

Residual Risk Level
2.0 - Low Impact / Low Probability

Stage
Identified

4.35 **Data loss causing damage or distress to individuals or damage the University's business.**
If this risk applies, click 'yes' and give a brief explanation of why the risk applies.

If the risk does not apply, click 'no'

Response
No

Justification
None

4.38 **Despite proper security, is there an increased possibility of external unlawful access to the data such as hacking?**
If this risk applies, click 'yes' and give a brief explanation of why the risk applies.

If the risk does not apply, click 'no'

Response
No

Justification
None

4.41 **Using an external data processor or sharing with another data controller increases the risk of unlawful access to personal data.**
If this risk applies, click 'yes' and give a brief explanation of why the risk applies.

If the risk does not apply, click 'no'

Response
No

Justification
None

4.47 **Any other risk you have identified - describe below.**
If this risk applies, click 'yes' and give a brief explanation of why the risk applies.

If the risk does not apply, click 'no'

Response
No

Justification
None

4.50 **Any other risk you have identified - describe below.**
If this risk applies, click 'yes' and give a brief explanation of why the risk applies.

If the risk does not apply, click 'no'

Response
No

Justification
None

4.53 **Any other risk you have identified - describe below.**
If this risk applies, click 'yes' and give a brief explanation of why the risk applies.

If the risk does not apply, click 'no'

Response
No

Justification
None

6 Submit

6.1 **Submit**
Please now click the blue 'Submit' button in the bottom right corner.

Supplemental Tables

Table S1. Type 2 myocardial infarction criteria and etiology of supply-demand imbalance in patients with an adjudicated diagnosis of type 2 myocardial infarction according to coronary artery disease

	Overall	***No coronary artery disease***	***Coronary artery disease***
**Number of participants**	93	30	63
Symptoms of ischemia	79 (85)	26 (87)	53 (84)
Sings of ischemia	71 (76)	22 (73)	49 (78)
Primary etiology of supply-demand imbalance			
**Coronary**	18 (19)	9 (30)	9 (14)
Coronary dissection	6 (7)	5 (17)	1 (2)
Coronary embolism	7 (8)	3 (10)	4 (6)
Coronary vasospasm	5 (5)	1 (3)	4 (6)
**Systemic**	30 (32)	6 (20)	24 (38)
Anemia	8 (9)	1 (3)	7 (11)
Hypotension	5 (5)	1 (3)	4 (6)
Hypoxemia	10 (11)	2 (7)	8 (13)
Severe hypertension	7 (8)	2 (7)	5 (8)
**Arrhythmia**	45 (48)	15 (50)	30 (48)
Bradyarrhythmia	1 (1)	0 (0)	1 (2)
Tachyarrhythmia	44 (47)	15 (50)	29 (46)
Number of supply-demand imbalance etiologies			
Single	76 (82)	24 (80)	52 (83)
Multiple	17 (18)	6 (20)	11 (17)

Number (%)

Table S2. Baseline characteristics for DEMAND-MI study participants and High-STEACS[13,14] trial population with an adjudicated diagnosis of type 2 myocardial infarction

	DEMAND-MI type 2 myocardial infarction population	***High-STEACS type 2 myocardial infarction population***
**Number of participants**	93	1,121
Age (years)	66 [55, 75]	74 [63, 87]
Female	41 (44)	620 (55)
Primary presenting symptoms		
Chest pain	65 (70)	749 (73)
Dyspnea	7 (8)	372 (25)
Palpitations	9 (10)	67 (6)
Syncope	10 (11)	38 (4)
Other	2 (2)	61 (6)
Past medical history		
Diabetes mellitus	11 (12)	147 (13)
Myocardial infarction	14 (15)	163 (15)
Coronary artery disease	26 (28)	454 (40)
Cerebrovascular disease	4 (4)	135 (12)
Heart failure	3 (3)	292 (26)
Previous revascularization		
PCI	10 (11)	97 (9)
CABG surgery	8 (9)	32 (3)
Medications at presentation		
Aspirin	25 (27)	471 (42)
Lipid lowering therapy	35 (38)	632 (56)
Beta-blocker	30 (32)	489 (44)
ACE Inhibitor or ARB	39 (42)	514 (56)
Oral anticoagulant	16 (17)	170 (15)

Median [interquartile range], number (%). Abbreviations: ACE=angiotensin-converting enzyme; ARB=angiotensin receptor blocker; CABG=coronary artery bypass graft; PCI=percutaneous coronary intervention.

Table S3. Baseline characteristics for study participants with an adjudicated diagnosis of type 2 myocardial infarction according to coronary imaging modality (coronary computed tomography angiography [CCTA] or invasive coronary angiography [ICA])

	Overall	*Invasive coronary angiography*	*Coronary CT angiography*	***P-value*
**Number of participants**	93	55	38	
Age (years)	66 [55, 75]	63 [53, 74]	69 [58, 76]	0.100
Female	41 (44)	24 (44)	17 (45)	>0.900
Current or previous cigarette smoker	14 (15)	10 (18)	4 (11)	0.500
Primary presenting symptoms				0.800
Chest pain	65 (70)	40 (73)	25 (66)	
Dyspnea	7 (8)	3 (6)	4 (11)	
Palpitations	9 (10)	5 (9)	4 (11)	
Syncope	10 (11)	6 (11)	4 (11)	
Other	2 (2)	1 (2)	1 (3)	
Past medical history				
Diabetes mellitus	11 (12)	4 (7)	7 (18)	0.120
Hypercholesterolemia	17 (18)	10 (18)	7 (18)	>0.900
Hypertension	40 (43)	23 (42)	17 (45)	>0.900
Myocardial infarction	14 (15)	7 (13)	7 (18)	0.600
Cerebrovascular disease	4 (4)	2 (4)	2 (5)	>0.900
Atrial fibrillation	16 (17)	6 (11)	10 (26)	0.100
Heart failure	3 (3)	2 (4)	1 (3)	>0.900
Chronic obstructive pulmonary disease	10 (11)	7 (13)	3 (8)	0.500
Other chronic respiratory illness	9 (10)	6 (11)	3 (8)	0.700
Malignancy	10 (11)	5 (9)	5 (13)	0.700
Previous revascularization				
PCI	10 (11)	5 (9)	5 (13)	0.700
CABG surgery	8 (9)	3 (6)	5 (13)	0.300
Medications at presentation				
Aspirin	25 (27)	13 (24)	2 (32)	0.500
P2Y12 receptor antagonist	13 (14)	10 (18)	3 (8)	0.300
Lipid lowering therapy	35 (38)	20 (36)	5 (39)	>0.900
Beta-blocker	30 (32)	14 (25)	16 (42)	0.140
ACE Inhibitor or ARB	39 (42)	22 (40)	17 (45)	0.800
Nitrates	22 (24)	11 (20)	11 (29)	0.500
Oral anticoagulant	16 (17)	6 (11)	10 (26)	0.100

Median [interquartile range], number (%). Abbreviations: ACE=angiotensin-converting enzyme; ARB=angiotensin receptor blocker; CABG=coronary artery bypass graft; IQR=interquartile range; PCI=percutaneous coronary intervention.
**Between-group comparisons are Fisher's exact test; or Wilcoxon rank-sum test.*

Table S4. Findings on coronary imaging according to study investigation - invasive coronary angiography or coronary computed tomography angiography (functional assessment and plaque characteristics)

	Invasive coronary angiography	*Coronary CT angiography*
**Number of participants**	55	38
OCT performed	7 (13)	-
Total number of lesions OCT performed on	9	-
Evidence of plaque rupture on OCT	1 (14)	-
Invasive FFR available	9 (10)	-
Invasive FFR value	0.85 [0.75, 0.93]	-
Invasive FFR with evidence of obstruction	4 (7)	-
FFR-CT available	-	15 (39)
FFR-CT value	-	0.83 [0.71, 0.88]
FFR-CT with evidence of obstruction	-	9 (24)
CT atherosclerotic burden and plaque characteristics		
Total calcium score, Agatston units	-	76 [0, 304]
Total plaque burden, %	-	35 [29, 46]
Non-calcified plaque burden, %	-	34 [29, 40]
Calcified plaque burden, %	-	1.6 [0.2, 3.7]
Low attenuation plaque burden, %	-	1.64 [0.89, 2.28]

Median [interquartile range], number (%). Abbreviations: CT=computed tomography; FFR=fractional flow reserve; OCT=optical coherence tomography.
[§]*Proportion (%) of number of patients who underwent OCT.*

Table S5. Findings on cardiac magnetic resonance imaging

	Cardiac magnetic resonance imaging
**Number of participants**	72
Left ventricular volumes and mass	
LVEDVI, mL/m^2	73 [61, 90]
LVESVI, mL/m^2	28 [21, 43]
LV mass index, g/m^2	74 [57, 84]
T2 mapping available	62 (67)
T2 value (septum, no LGE present) ms	39.6 [37.9, 41.6]
Late gadolinium sequence performed	68 (94)
LGE present	37 (54)
LGE pattern§	
Infarct LGE	33 (49)
Subendocardial	22 (32)
Transmural	11 (16)
Single - territory	31 (40)
Multi - territory	2 (3)
T2 value (infarct-pattern LGE) ms	48.9 [43.9-54.2]
Non-ischemic LGE	4 (6)
Epicardial	1 (1)
Mid-wall	3 (4)
Non-ischemic LGE with T2 mapping available	2 (3)
Acute (high T2 value)	1 (1)
Old (normal T2 value)	1 (1)

Median [interquartile range], number (%). Abbreviations: LGE=late gadolinium enhancement; LV=left ventricle; LVEDVI=left ventricular end-diastolic volume index; LVESVI=left ventricular end-systolic volume index.
§ Proportion (%) of the number of patients with available late gadolinium sequences

Table S6. Etiology of supply-demand imbalance in type 2 myocardial infarction according to imaging evidence of type 2 myocardial infarction

	No imaging evidence of myocardial infarction	***Imaging evidence of myocardial infarction***
**Number of participants**	54	39
**Coronary**	4 (10)	14 (36)
Coronary dissection	2 (5)	4 (10)
Coronary embolism	1 (3)	6 (15)
Coronary vasospasm	1 (3)	4 (10)
**Systemic**	17 (44)	13 (33)
Anemia	6 (15)	2 (5)
Hypotension	2 (5)	3 (8)
Hypoxemia	5 (13)	5 (13)
Severe hypertension	4 (10)	3 (8)
**Arrhythmia**	33 (85)	12 (30)
Bradyarrhythmia	1 (3)	0 (0)
Tachyarrhythmia	32 (82)	12 (30)
Number (%)		

Table S7. Baseline characteristics for study participants with an adjudicated diagnosis of type 2 myocardial

	No abnormality on cardiac and coronary imaging	*Abnormality on cardiac or coronary imaging*	**P-value*
**Number of participants**	10	83	
Age (years)	60 [53, 67]	67 [56, 75]	0.200
Female	8 (80)	33 (40)	0.020
Current or previous cigarette smoker	5 (50)	33 (40)	0.700
Primary presenting symptoms			0.600
Chest pain	7 (70)	58 (70)	
Dyspnea	1 (10)	6 (7)	
Palpitations	0 (0)	9 (11)	
Syncope	2 (20)	8 (10)	
Other	0 (0)	2 (3)	
Past medical history			
Diabetes mellitus	0 (0)	11 (13)	0.600
Hypercholesterolemia	0 (0)	17 (20)	0.200
Hypertension	3 (30)	37 (45)	0.500
Myocardial infarction	0 (0)	14 (17)	0.300
Cerebrovascular disease	0 (0)	4 (5)	>0.900
Atrial fibrillation	1 (10)	15 (18)	>0.900
Heart failure	0 (0)	3 (4)	>0.900
Chronic obstructive pulmonary disease	2 (20)	8 (10)	0.300
Other chronic respiratory illness	1 (10)	8 (10)	>0.900
Malignancy	0 (0)	10 (12)	0.600
Previous revascularization			
PCI	0 (0)	10 (12)	0.600
CABG surgery	0 (0)	8 (10)	0.600
Medications at presentation			
Aspirin	0 (0)	25 (30)	0.057
P2Y12 receptor antagonist	0 (0)	13 (16)	0.300
Lipid lowering therapy	0 (0)	35 (42)	0.012
Beta-blocker	1 (10)	29 (35)	0.200
ACE Inhibitor or ARB	2 (20)	37 (45)	0.200
Nitrates	0 (0)	22 (27)	0.110
Oral anticoagulant	0 (0)	16 (19)	0.200
Investigations at presentation			
Peak hs-cTnI, ng/L	966 [266, 1,792]	1,277 [1,165, 4,046]	0.300
Etiology of supply-demand imbalance			0.800
Coronary dissection	0 (0)	6 (7)	
Coronary embolism	0 (0)	7 (8)	
Coronary vasospasm	0 (0)	5 (6)	
Anemia	1 (10)	7 (8)	
Hypotension	1 (10)	4 (5)	

Hypoxemia	2 (20)	8 (10)
Severe hypertension	0 (0)	7 (8)
Bradyarrhythmia	0 (0)	1 (1)
Tachyarrhythmia	6 (60)	38 (46)

Median [interquartile range], number (%). Abbreviations: ACE=angiotensin-converting enzyme; ARB=angiotensin receptor blocker; CABG=coronary artery bypass graft; PCI=percutaneous coronary intervention.
**Between-group comparisons are Fisher's exact test; or Wilcoxon rank-sum test.*

Table S8. Individual patient data for patients with normal coronary and cardiac imaging

Age	*Gender*	*Peak high-sensitivity cardiac troponin I (ng/L)*	*Presenting symptoms*	*Admission electrocardiogram findings*	*Etiology of supply-demand imbalance*	*Clinical diagnosis*	*Risk factors*
80-90	Male	2,197	Breathlessness	Atrial fibrillation, new T wave inversion (lateral leads)	Hypoxemia, Tachyarrhythmia	Type 2 respiratory failure in the context of exacerbation of chronic obstructive disease	Hypertension, Smoker
50-60	Male	240	Cardiac arrest with preceding chest pain	Ventricular fibrillation with sinus rhythm and new left bundle branch block following resuscitation	Tachyarrhythmia, Hypotension	Out of hospital cardiac arrest (normal coronary angiogram and cardiac MRI)	Non-smoker
70-80	Female	206	Palpitations, syncope	Sinus rhythm, new ST segment depression (lateral leads)	Hypotension, Tachyarrhythmia	Atrial fibrillation, pulmonary embolus	Hypertension, Non-smoker
50-60	Female	1,364	Chest pain, palpitations	Sinus rhythm, new ST segment depression (anterior leads)	Tachyarrhythmia	Paroxysmal supraventricular tachycardia	Ex-smoker
60-80	Female	1,841	Chest pain, breathlessness	Sinus rhythm, new T wave inversion (anterior leads)	Hypoxemia	Infective exacerbation of COPD	Ex-smoker
30-40	Female	61	Chest pain	Sinus rhythm, new T wave inversion (anterior leads)	Anemia	Upper gastrointestinal bleed secondary to a duodenal ulcer	Hypertension Smoker
30-40	Female	67	Chest pain, palpitations	Sinus rhythm	Tachyarrhythmia	Paroxysmal supraventricular tachycardia	Non-smoker
60-70	Female	1,647	Chest pain, palpitations	Supraventricular tachycardia, new T wave inversion (lateral leads)	Tachyarrhythmia	Paroxysmal supraventricular tachycardia	Non-smoker
50-60	Female	342	Chest pain, palpitations	Sinus rhythm	Tachyarrhythmia	Paroxysmal supraventricular tachycardia	Ex-smoker
60-70	Female	567	Chest pain	Sinus rhythm	Tachyarrhythmia	Atrioventricular nodal re-entry tachycardia	Non-smoker

Supplemental Figures

Figure S1. DEMAND-MI case with adjudicated diagnosis of type 1 myocardial infarction due to plaque rupture on optical computed tomography (OCT).

(Abbreviations: LAD=left anterior descending coronary artery)

Patient in their 80s admitted to hospital with shortness of breath and cough. Patient experienced worsening angina over a two weeks period prior to admission. Clinical examination on admission revealed signs of infection and following further investigations he was started on treatment for confirmed pneumonia. Patient underwent coronary angiography, which showed severe proximal left anterior coronary artery disease (A) and complete total occlusion of the proximal right coronary artery. Optical computed tomography showed evidence of plaque rupture (B) in the proximal LAD. Cardiac magnetic resonance imaging revealed evidence of anteroseptal wall motion abnormality and corresponding subendocardial late gadolinium enhancement.

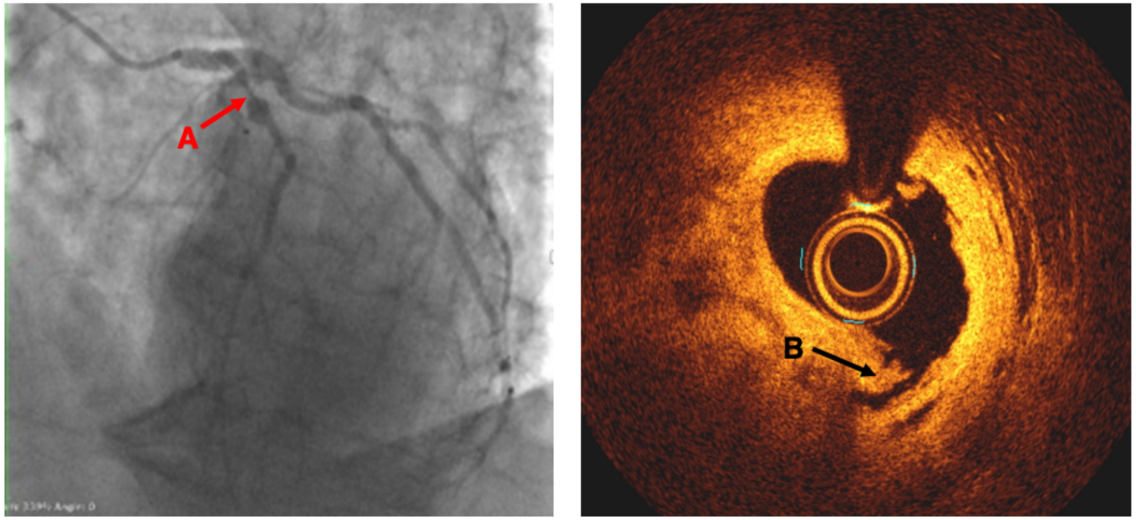

Figure S2. Cardiac magnetic resonance imaging showing multi-territory late gadolinium enhancement in three study participants

Infarct pattern multi-territory late gadolinium enhancement

Patient in their 60s admitted with sudden onset chest pain. They had a previous anterior ST-elevation myocardial infarction. Admission electrocardiogram showed slow new atrial fibrillation with inferior ST segment elevation. Emergency coronary angiography revealed abrupt distal occlusion of the right coronary artery due to multiple emboli. Cardiac MRI revealed sub-endocardial late gadolinium enhancement of the anterior wall (A) and apex (B) consistent with previous infarct. Large island of microvascular obstruction (MVO) was noted in the basal to mid inferior and inferolateral walls (C) consistent with recent right coronary artery territory infarct.

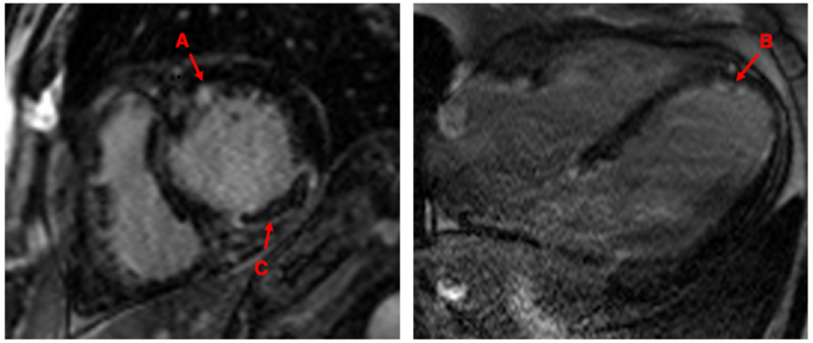

Patient in their 50s admitted to hospital following an episode of collapse preceded by palpitations. Admission electrocardiogram showed supraventricular tachycardia and evidence of global ischemia. Invasive coronary angiography was unremarkable and showed normal coronary arteries. Patient underwent cardiac magnetic resonance imaging, which showed evidence of basal sub-endocardial late gadolinium enhancement of the inferior and inferolateral walls (A), with a second discrete area affecting the anterior wall (B).

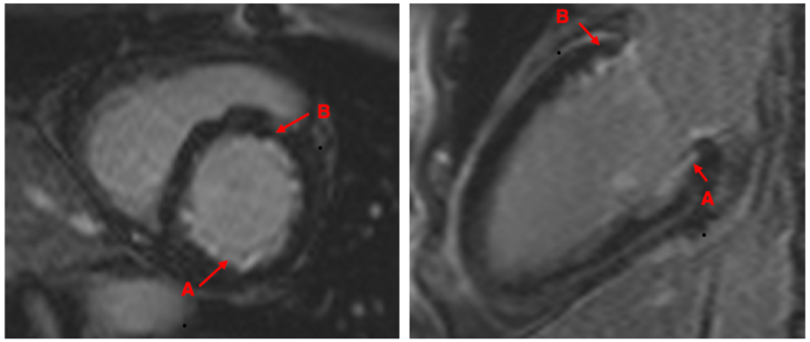

Non-ischemic pattern multi-territory late gadolinium enhancement

Patient in their 50s admitted to hospital with chest pain and palpitations. Patient did not have any significant past medical history. Patient underwent coronary angiography, which showed normal appearance of the coronary arteries. Cardiac magnetic resonance imaging revealed extensive epicardial (A) and mid-wall (B) fibrosis throughout the basal and mid-ventricle, consistent with non-ischemic cardiomyopathy.

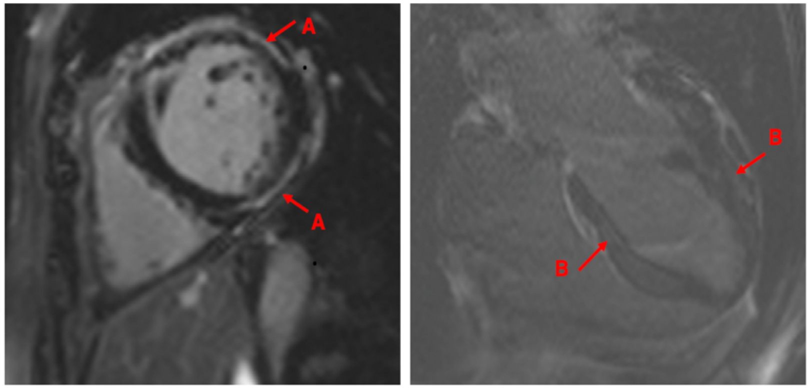

Figure S3. Cardiac magnetic resonance imaging showing patterns of structural heart disease in patients with an adjudicated diagnosis of type 2 myocardial infarction

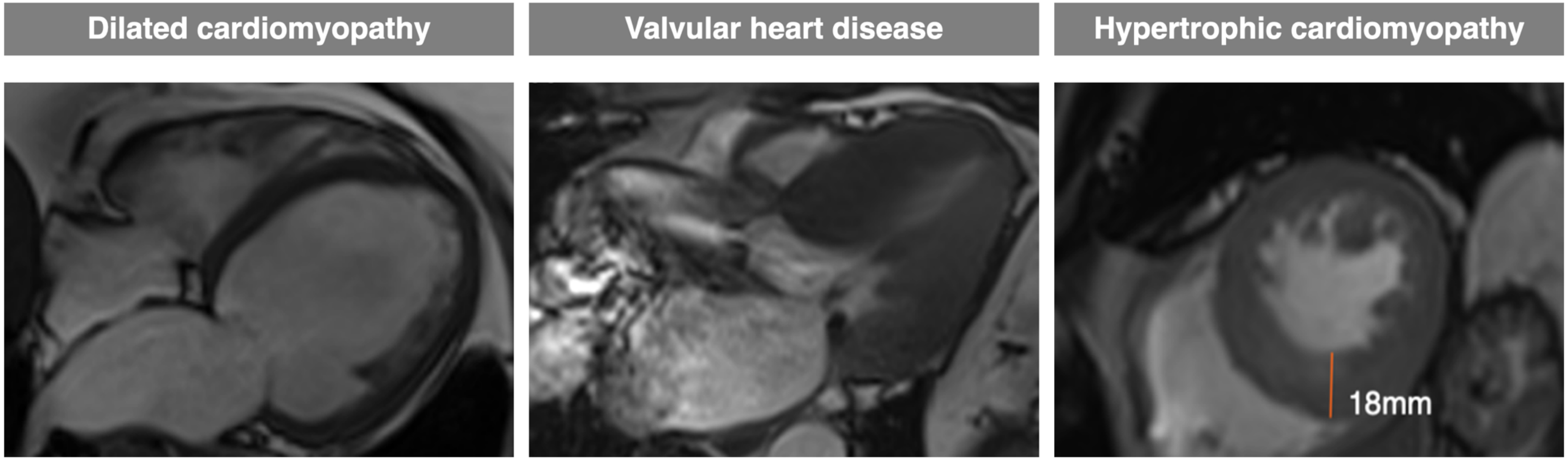

Figure S4. Panel of alluvial plots evaluating the implication of study findings on clinical management in patients with type 2 myocardial infarction (N=93) according to identified coronary disease (A) or left ventricular impairment (B). Data show proportion of patients (%) with identified findings and management implications.

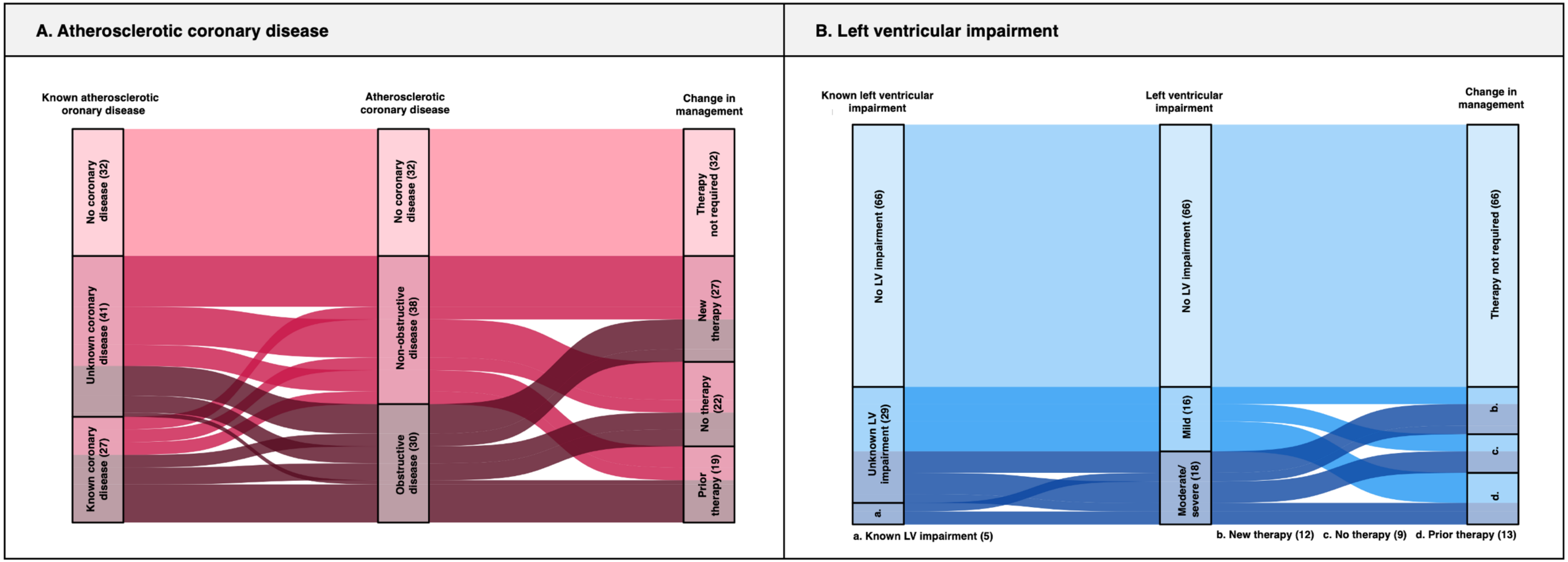

***De*termining the *me*chanism of myocardial injury *and* role of coronary disease in type 2 *M*yocardial *I*nfarction:**

DEMAND-MI

Co-sponsor	**The University of Edinburgh and Lothian Health Board<br>ACCORD<br>The Queen's Medical Research Institute<br>47 Little France Crescent<br>Edinburgh<br>EH16 4TJ**
Protocol authors	**Dr Andrew Chapman, Dr Scott Semple, Prof Edwin van Beek, Dr Marc Dweck, Prof Nicholas Mills, Prof David Newby,**
Funder	**British Heart Foundation**
Funding Reference Number	**FS/16/75/32533**
Chief Investigator	**Dr Andrew Chapman, Clinical Research Fellow**
Sponsor number	**AC17042**
REC Number	**17/SS/0078**
Project registration	**NCT03338504**
Version Number and Date	**Version 2.5 19th December 2019**

Trial Start Date: 1st August 2017
Trial Finish Date: 1st August 2020
Trial Report Date: 1st November 2020

**Amendment classification and number:**	**Summary of change(s)**
Version 2	Updated trial start and finish dates<br>Detailed demographic data to be recorded<br>Updated consent information<br>Included patient preference for coronary angiography modality<br>Detailed the genetic analysis to be undertaken
Version 2.1	Inclusion of stress-perfusion MRI
Version 2.2	Updated exclusion criteria
Version 2.3	Addition of serum sample tube to biomarker sampling
Version 2.4	Revised title and clarified inclusion criteria
Version 2.5	Updated recruitment and exclusion criteria

COORDINATING CENTRE

**Chief Investigator**	**Co-Investigator**
Dr Andrew R Chapman<br>Room SU305, Chancellor's Building,<br>University of Edinburgh, Royal Infirmary,<br>Little France, Edinburgh, EH16 4SB, UK.<br><br>Tel: +44 (0) 131 242 6517<br>Fax: +44 (0) 131 242 6379<br>Email: a.r.chapman@ed.ac.uk	Professor Nicholas L Mills<br>Room SU225, Chancellor's Building,<br>University of Edinburgh, Royal Infirmary,<br>Little France, Edinburgh, EH16 4SB, UK.<br><br>Tel: +44 (0) 131 242 6517<br>Fax: +44 (0) 131 242 6379<br>Email: nick.mills@ed.ac.uk
**Co-Investigator**	**Co-Investigator**
Professor David E Newby<br>Room SU225, Chancellor's Building,<br>University of Edinburgh, Royal Infirmary,<br>Little France, Edinburgh, EH16 4SB, UK.<br><br>Tel: +44 (0) 131 242 6517<br>Fax: +44 (0) 131 242 6379<br><br>Email: d.e.newby@ed.ac.uk	Dr Marc Dweck<br>Room SU225, Chancellor's Building,<br>University of Edinburgh, Royal Infirmary,<br>Little France, Edinburgh, EH16 4SB, UK.<br><br>Tel: +44 (0) 131 242 6517<br>Fax: +44 (0) 131 242 6379<br><br>Email: marc.dweck@ed.ac.uk
**Co-Investigator**	**Co-Investigator**
Professor Edwin Van Beek<br>Edinburgh Imaging Facility, QMRI,<br>47 Little France Crescent, Edinburgh,<br>EH16 4TJ, UK.<br><br>Tel: +44 (0) 131 242 7660<br>Fax: +44 (0) 131 242 7773<br><br>Email: edwin-vanbeek@ed.ac.uk	Dr Scott Semple<br>Clinical Research Imaging Centre,<br>47 Little France Crescent, Edinburgh,<br>EH16 4TJ, UK.<br><br>Tel: +44 (0) 131 242 7660<br>Fax: +44 (0) 131 242 7773<br><br>Email: scott.semple@ed.ac.uk

PARTICIPATING SITES

**NHS Lothian**<br>**(Royal Infirmary of Edinburgh)**<br>**Chief Investigator**<br>Dr Andrew R Chapman<br>Room SU305, Chancellor's Building, University of Edinburgh, Royal Infirmary, Little France, Edinburgh, EH16 4SB, UK.<br>Tel: +44 (0) 131 242 6517<br>Fax: +44 (0) 131 242 6379<br>Email: a.r.chapman@ed.ac.uk	**NHS Lothian**<br>**(Royal Infirmary of Edinburgh)**<br>**Co-Investigator / Supervisor**<br>Professor Nicholas L Mills<br>Room SU225, Chancellor's Building, University of Edinburgh, Royal Infirmary, Little France, Edinburgh, EH16 4SB, UK.<br>Tel: +44 (0) 131 242 6517<br>Fax: +44 (0) 131 242 6379<br>Email: nick.mills@ed.ac.uk

Contents:

Section		Pages
1.	Protocol Approval	6
2.	Investigator Statement	7
3.	Summary	8
4.	Background	9
5.	Study Objectives	10
6.	Trial Design	13
7.	Study Procedures	15
8.	Statistical Analysis	17
9.	Expected Results	17
10.	Safety Reporting and Study Monitoring	18
11.	Oversight Arrangements	20
12.	Good Clinical Practice	21
13.	Study Conduct Responsibilities	23
14.	References	24
15.	Appendices	25

1 Protocol Approval

Determining the mechanism of myocardial injury and role of coronary disease in type 2 Myocardial Infarction

Signatures

Dr Andrew Chapman<br>Chief Investigator	____________________<br>Signature	____________<br>Date
Prof Nicholas Mills<br>Co-Investigator	____________________<br>Signature	____________<br>Date

2 Investigator Statement

Determining the mechanism of myocardial injury and role of coronary disease in type 2 Myocardial Infarction

I agree to conduct the study according to this protocol, the principles of International Conference on Harmonisation Tripartite Guidelines for Good Clinical Practice (ICH GCP) and the applicable regulatory requirements. Any changes in procedure will only be made if necessary to protect the safety, rights or welfare of the patients.

I agree to take responsibility for the conduct of the study and ensure that all other staff involved are adequately informed about the protocol and amendments and their study related duties and functions.

Signatures

_________________________________ ______________

Signature of Investigator Date

____

Name of Investigator (please print)

3 Summary

Myocardial injury is common in patients without acute coronary syndrome, and therefore international guidelines propose a classification of patients with myocardial infarction by aetiology. This differentiates between myocardial infarction due to plaque rupture (type 1) and myocardial oxygen supply-demand imbalance (type 2) in other acute illnesses. However, these guidelines have not been widely adopted as the diagnostic criteria for type 2 myocardial infarction are not clearly defined. Patients with type 2 myocardial infarction have poor long term outcomes, with at least twice the mortality at five years compared to those with an index type 1 myocardial infarction. Despite the majority of deaths being attributable to non-cardiovascular events, the rate of future type 1 myocardial infarction or cardiovascular death is similar regardless of index classification. If this future risk is related to the presence of underlying coronary artery disease, then there may be the potential to improve outcomes through targeted investigation and secondary prevention. We will undertake a systematic evaluation of the mechanism of myocardial injury and the role of coronary artery disease in 100 patients with elevated cardiac troponin concentrations where the diagnosis is likely to be type 2 myocardial infarction. These studies will help improve the assessment of patients with myocardial injury, refine the diagnostic criteria for type 2 myocardial infarction, and aid the design of future therapeutic trials.

4 Background

The definition of acute myocardial infarction has evolved to accommodate increasingly sensitive markers of myocardial necrosis and imaging methods that allow greater understanding of the pathogenic mechanisms of acute coronary syndrome. As such, the universal definition of myocardial infarction now proposes that we classify patients with myocardial infarction based on aetiology.(1) Whilst this classification has been used in clinical trials to refine clinical outcomes(2-4), it has not been widely adopted in clinical practice, and the frequency and implications of subtypes of acute myocardial infarction are uncertain. We believe the diagnostic criteria for type 2 myocardial infarction require clarification and that this is necessary to encourage clinicians to adopt the proposed classification. This can only be achieved through prospective and systematic evaluation of the clinical presentation, pathophysiological mechanisms and outcomes of unselected patients with acute myocardial injury in clinical practice.

Classification of myocardial infarction

The Universal Definition of Myocardial Infarction differentiates between type 1 myocardial infarction due to thrombosis of an atherosclerotic plaque, and type 2 myocardial infarction due to an imbalance in myocardial oxygen supply and demand in another acute illness.(1) The classification describes evidence of myocardial necrosis in the absence of myocardial ischemia as myocardial injury. Myocardial infarction presenting as sudden death (type 3), or after percutaneous coronary intervention (type 4) and coronary artery bypass grafting (type 5) are also defined. This classification is contentious and was based on expert consensus rather than evidence from prospective clinical trials. The most controversial diagnosis is that of type 2 myocardial infarction as these patients are heterogeneous, and have myocardial ischemia secondary to a wide range of primary acute medical or surgical conditions. It is here where the diagnosis of type 2 myocardial infarction has the greatest potential for benefit and for harm. For instance, a patient with type 2 myocardial infarction secondary to a tachyarrhythmia may be identified as having three vessel coronary artery disease and undergo surgical revascularisation. In contrast, the inappropriate use of anti-platelet agents and anti-coagulants in a patient with myocardial ischemia due to hypotension and occult gastrointestinal bleeding is likely to accelerate bleeding and may be fatal.

The global task force are reviewing the Universal Definition of Myocardial Infarction and recognise the need to provide clearer diagnostic criteria and guidance.(5) Based on the current guideline the differentiation between patients with type 2 myocardial infarction and myocardial injury is subjective and therefore inconsistent in clinical practice.(6,7) Likewise, in the absence of an accepted definition it is difficult to conduct randomised trials to determine the effectiveness of secondary prevention, such as aspirin or statins.

Incidence of type 2 myocardial infarction and myocardial injury in clinical practice

Following improvements in assay performance, we introduced a more sensitive troponin assay at our institution.(8,9) The validation and subsequent implementation of this assay provided an opportunity to assess the impact of lowering the diagnostic threshold on the incidence, management and clinical outcome of patients with type 2 myocardial infarction and myocardial injury.(10) We systematically evaluated all patients with elevated plasma troponin concentrations ***irrespective of their presenting complaint*** who were admitted to the

Royal Infirmary of Edinburgh during the validation and implementation of a sensitive cardiac troponin I assay (n=2,122) and made a number of novel observations. First, type 2 myocardial infarction and myocardial injury are as common as type 1 myocardial infarction in clinical practice. The incidence of type 2 myocardial infarction or myocardial injury increases with age and is more common than type 1 myocardial infarction in patients ≥75 years of age. Lowering the diagnostic threshold preferentially increased the number of patients identified with type 2 myocardial infarction or myocardial injury. Indeed, for every additional patient reclassified as type 1 myocardial infarction, we identified three patients with type 2 myocardial infarction or myocardial injury (257 versus 672 patients, P<0.001).[10] This is important as the advent of newer highly sensitive cardiac troponin assays with lower thresholds may identify an even higher proportion of patients with previously undetectable myocardial injury.

Long term outcomes in patients with type 2 myocardial infarction and myocardial injury

Patients with type 2 myocardial infarction have poor outcomes,[11-15] worse than those with type 1 myocardial infarction, with 62.5% (268/429) versus 36.7% (430/1,171) dead at five years (adjusted hazard ratio [HR] 1.36, 95%CI 1.16-1.59). Survival in patients with myocardial injury is worse, even compared to those with type 2 myocardial infarction with 72.4% (378/522) dead at five years (HR 1.25, 95%CI 1.07-1.46 [***Figure 1 - below***]***;*** manuscript in preparation).

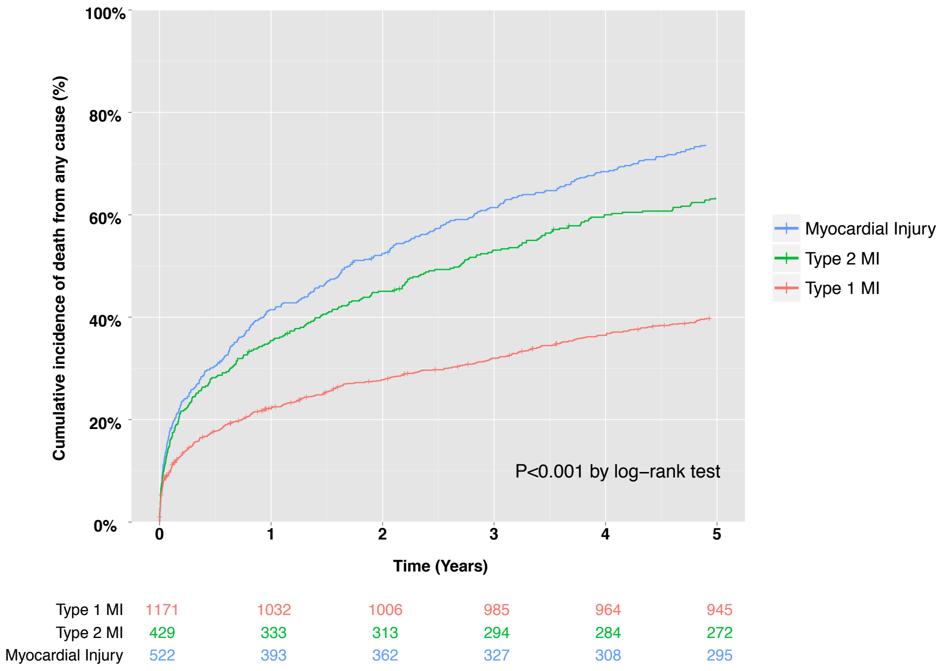

Despite the excess in five-year mortality being largely attributable to non-cardiovascular death, the risk of future major adverse cardiovascular events (MACE; future type 1 myocardial infarction or cardiovascular death) is similar, regardless of index diagnosis. We found patients with type 2 myocardial infarction or myocardial injury and known coronary artery disease

were at significantly increased risk of MACE, yet the majority of patients did not receive invasive coronary investigation nor optimal secondary prevention [***Figure 2 - below***].

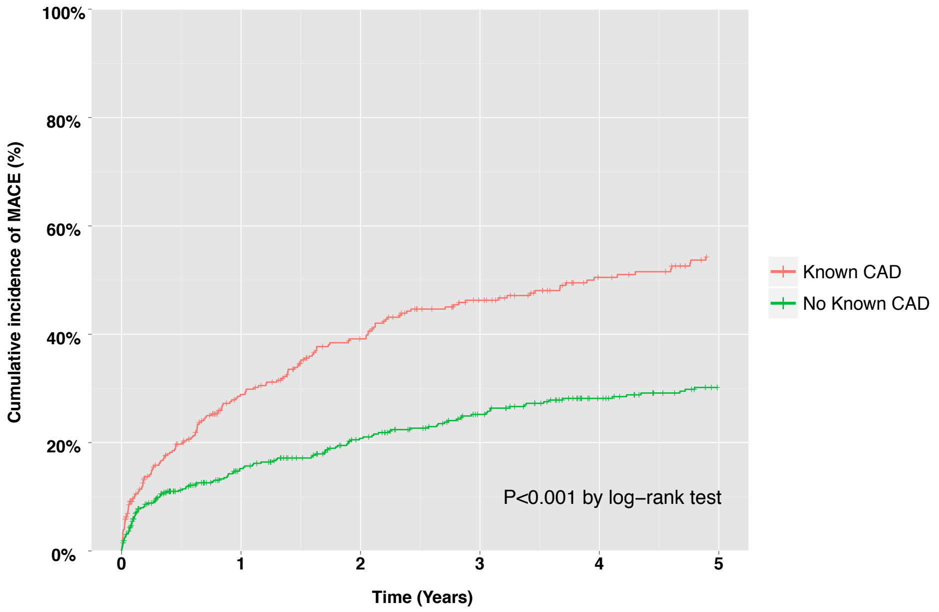

Indeed, given patients with type 2 myocardial infarction are older and have a higher prevalence of co-morbidities, it is likely that there is an unrecognised burden of previously clinically quiescent coronary artery disease. It is here where there may be the opportunity to improve patient outcomes through identification and targeted secondary prevention.

5 Study Objectives

We propose to systematically evaluate the mechanisms of acute myocardial injury in unselected patients who present to hospital with an alternative primary illness likely to cause myocardial oxygen supply or demand imbalance. All patients will be assessed by a member of the study team during their index admission and will undergo a detailed assessment of their coronary anatomy with either computed tomography coronary angiography (CTCA), CT calcium scoring and non-invasive fractional flow reserve assessment (CT-FFR) *or* invasive coronary angiography with optical coherence tomography (OCT) and invasive fractional flow reserve (FFR). The pattern of myocardial injury and its functional consequence will be evaluated by cardiac magnetic resonance (CMR) imaging. We will determine the kinetics of cardiac troponin release using serial testing at multiple time points throughout admission, and quantify other proteins and the expression of long non-coding RNA and associated mRNA to identify differences related to the presence of coronary artery disease, which may help to identify new biomarkers.

Understanding the mechanisms of acute myocardial injury in hospitalised patients and the contribution of coronary artery disease will lead to the development of a diagnostic algorithm and framework for clinicians to base their assessment, and will help to guide future therapeutic trials.

5.1 Original Hypothesis

The majority of patients with myocardial injury secondary to oxygen supply or demand imbalance will have evidence of underlying coronary artery disease.

6 Trial Design

Design: Prospective cohort study

Setting: Royal Infirmary of Edinburgh, a tertiary cardiac centre

Study population:
We will identify consecutive patients with acute myocardial injury (defined as a rise and or fall in cardiac troponin concentration on serial testing, with at least one value >99th centile) where the likely mechanism of injury is thought to be myocardial oxygen supply and demand imbalance (e.g secondary to hypoxia, hypotension, tachycardia or anaemia). Patients will be identified through screening of cardiac troponin measurements using the electronic patient record and laboratory databases at the recruiting site. The chief investigator is a clinical research fellow and honorary cardiology registrar in NHS Lothian, and has access to electronic patient records as part of routine clinical care. All patients screened will be recorded in a screening log. In patients that meet our inclusion criteria, but have one or more exclusion criteria and therefore are not eligible for enrolment, we will record demographic and clinical information from the electronic patient record with approval from the local Caldicott Guardian (including age, gender, previous medical history such as hypertension, diabetes, stroke, angina, myocardial infarction, previous angioplasty or bypass surgery, medication history, presenting complaint and ECG findings). Patients who meet both the inclusion and exclusion criteria, will be approached and those who provide consent will comprise the study population and be allocated a unique study number.

Number of participants:
We will recruit one hundred patients from the Royal Infirmary of Edinburgh, Scotland.

Inclusion criteria:
- Unscheduled hospital admission with acute myocardial injury (defined as a rise and or fall in high-sensitivity cardiac troponin I concentrations on blood testing)
- A suspected aetiology of myocardial oxygen supply and demand imbalance with symptoms or signs of myocardial ischaemia

Exclusion criteria:
- Unable or unwilling to give informed consent
- Women who are pregnant or breastfeeding will not be enrolled into the trial.
- Probable type 1 myocardial infarction
- Renal impairment (estimated glomerular filtration rate ≤30ml/min/1.73m^2)
- Severe hepatic impairment
- Frailty with inability to self-transfer (determined using Katz Index)

Primary objective:
- To determine the prevalence and severity of coronary artery disease (defined as stenosis >50% in a major epicardial vessel) in patients with myocardial injury

secondary to oxygen supply demand imbalance using invasive or CT coronary angiography

Secondary objectives:

- To determine the functional significance of coronary artery stenosis using the invasive or CT fractional flow reserve technique.
- To determine the prevalence of intraluminal plaque rupture using optical coherence tomography
- To evaluate the pattern of myocardial injury or infarction using the late gadolinium enhancement technique
- To determine the presence of myocardial ischaemia quantified using stress-perfusion magnetic resonance imaging.
- To validate a prediction model derived in patients with an adjudicated diagnosis of type 2 myocardial infarction identified in the High-STEACS clinical trial.
- To identify novel biomarkers that differentiate between type 1 and type 2 myocardial infarction
- To evaluate the relationship between coronary artery disease and cardiovascular outcomes

Consent

The chief investigator will screen all measurements of cardiac troponin I to identify patients with evidence of myocardial injury within the previous 24 hours, and after review of the electronic patient record will liaise other members of the clinical team to identify patients who may be suitable for recruitment. The clinical team will approach the patient to obtain verbal consent for the researcher to discuss the study. All patients will receive a patient information sheet at the time of screening and will be provided with an opportunity to ask questions. Eligible patients will be given a minimum of one hour to consider the written material and the investigator or another suitably qualified member of the research team will then re-attend to provide an opportunity for further questions. As patients may be recruited from the emergency department or the acute medical unit where there is potential for early discharge, a short time interval is necessary to permit recruitment prior to hospital discharge. Audit data shows it takes 124 minutes from arrival to troponin result at the Royal Infirmary of Edinburgh. Assuming a patient is identified as suitable within 30 minutes of sample result, this leaves only 90 minutes for screening and recruitment prior to possible discharge at four hours, in line with government standards. In women of child bearing age, a pregnancy test will be performed prior to recruitment. If a patient is identified as meeting inclusion criteria through screening but has already been discharged from hospital, the research team will liaise with the clinical care team who were responsible for the patients care. If the clinical care team agree the patient is a suitable candidate, the clinical care team will contact the patient by phone and offer to post a patient information leaflet to ensure they have an opportunity to consider the study. The patient information leaflet contains contact details for the research team and potential participants can then consider and get in touch with the research team directly to discuss and consent to the study. An independent study observer will be available to discuss any aspect of the study. Written informed consent will be obtained from all participants.

Withdrawal from study

Participation in this study is voluntary and subjects will be free to withdraw from study at any point should they wish to do so. Patients retain the right to ask for blood samples to be destroyed at any time.

7 Study Procedures

All participants will undergo cardiac magnetic resonance imaging of the myocardium where there are no contraindications, in addition to evaluation of coronary anatomy by either invasive or non-invasive coronary angiography dependent on their co-morbidities or patient preference (***Appendix 1*** – Flow Diagram). For example, frail patients or those with severe peripheral vascular disease in whom intra-vascular access may be challenging would be more likely to undergo non-invasive imaging. This clinical decision will be made in discussion with the consultant responsible for the patient's ongoing care by the investigators who are all cardiologists with experience of both invasive and non-invasive diagnostic imaging. Such investigations are often considered by the attending clinician as part of routine clinical care, but decisions are challenging given the lack of evidence to date. All patients will provide informed consent in line with routine clinical practice. We will report the results of all investigations to the patients attending clinician so therapy may be modified where this is felt to be of benefit. The target for completion of all diagnostic studies is 28 days from index presentation, with the aim to perform imaging as early as is feasible.

Cardiac Magnetic Resonance Imaging

Cardiovascular magnetic resonance (CMR) will be performed using a 3T scanner (MAGNETOM Verio, Siemens AG, Healthcare Sector, Erlangen, Germany) at the Clinical Research Imaging Centre (CRIC), Edinburgh, where there is established experience in research MRI. The MRI scan will consist of localisers, axial and coronal HASTE images, standard breath-held and ECG-gated cine sequences in 2 chamber, 4 chamber and short axis views. Short-axis cine images will be obtained using a balanced steady-state free precession sequence from the mitral valve annulus to the apex (8 mm parallel slices with 2 mm spacing) for the assessment of left ventricle function and volumes. Left ventricle volumes, mass and ejection fraction will be assessed using dedicated software (Argus Ventricular Function, Siemens AG Healthcare Sector, Erlangen, Germany) and values indexed to body surface area. Breath-held, ECG-gated T2 mapping sequences of the myocardium will be performed in the long-axis as a marker of myocardial inflammation. T1-weighted imaging of the coronary arteries will be performed to look for evidence of recent intraplaque thrombus or haemorrhage using the CATCH sequence. All patients will then receive 0.4mg (5ml) of peripheral Regadenoson (Rapiscan™) as a stress perfusion agent, and a bolus of 0.2 mmol/kg Gadolinium (Gadovist™), to quantify areas of myocardial ischaemia. Stress-perfusion imaging will not be undertaken in patients with severe asthma or chronic obstructive pulmonary disease, those on dipyridamole, theophylline or

aminophylline, or those with atrioventricular block. Patients will be asked to withhold caffeine intake for 12 hours prior to imaging. This will be followed by standard late-gadolinium enhancement sequences. The late gadolinium enhancement and T2 mapping techniques will identify regions of new or old myocardial infarction as well as other patterns of injury such as the mid-wall pattern associated with myocarditis.

Invasive coronary angiography and optical coherence tomography

Coronary angiography will be performed via the femoral or radial artery with 6F arterial catheters. In patients with one or more stenoses in a major epicardial vessel, a coronary pressure guidewire (PressureWire™ Aeris™, St. Jude Medical, St. Paul, Minnesota) will be used to determine distal coronary pressure and the fractional flow reserve (FFR) calculated at maximal adenosine-induced (intravenous 140 µg/kg/min) hyperaemia. As previously described, frequency domain optical coherence tomography (FD-OCT) will be performed in all three coronary vessels using a FastView® coronary imaging catheter (Terumo, Tokyo, Japan) with pullback at 20 mm/s to identify features consistent with vulnerable plaque or recent plaque rupture.[(16)] If there is evidence of inducible myocardial ischaemia due to coronary artery stenosis, revascularisation with percutaneous coronary intervention may be considered if in the patients best interests.

CT coronary angiography

CT coronary angiography will be performed in the Clinical Research Imaging Centre, according to previously published methodology.[(17)] Imaging will be performed using a 128 multidetector row CT (Siemens Biograph, Siemens Healthcare, Erlangen, Germany). Patients with a heart rate exceeding 65 beats/min will receive oral beta-blockade (50 or 100 mg metoprolol) 1 hour before computed tomography. Additional intravenous beta blockers will be given depending on heart rate at the time of imaging. All patients will receive sublingual glyceryl trinitrate (300 µg) immediately prior to dual cardiac and respiratory-gated computed tomography imaging of the coronary arteries. We will quantify total plaque burden using CT calcium scoring. A bolus of 80-100 mL of contrast (400 mg/mL; Iomeron, Bracco, Milan, Italy) will be injected intravenously at 5 mL/s. CT angiography will be evaluated jointly by a Radiologist and a Cardiologist with suitable training to determine the extent of coronary atherosclerosis. An assessment of the functional consequences of coronary artery stenosis will be made using the computed tomography fractional flow reserve (CT-FFR) technique, using the HeartFlow™ platform.[(18)]

High-sensitivity cardiac troponin I assay

Serial blood samples will be obtained on enrolment to the study, at 24 hours, and at last point of contact to the research team (on discharge from hospital or at outpatient visit for study imaging) in two 9 mL lithium-heparin tubes, two 9mL EDTA plasma tubes and two 9mL serum tubes. Samples obtained will facilitate development of novel biomarkers using proteomic and genomic approaches. The maximum sample volume obtained will be 180 ml. We will obtain wastage serum (surplus) from routinely obtained clinical samples. All blood samples will be stored at -80 degrees Celsius for future development, evaluation and audit of novel and existing cardiovascular biomarkers. High-sensitivity cardiac troponin I concentrations will be measured in batch processing using the ARCHITECT_{STAT} high-sensitive troponin I assay (Abbott Laboratories, Abbott Park, IL). This assay has a limit of detection of 1.2 ng/L and the inter-assay CV<10% at 4.7 ng/L. The upper reference limit (99th centile) is 26 ng/L, and is two-fold

higher in men (34 ng/L) than in women (16 ng/L).[19,20] All samples will be anonymised and linked by a unique non-identifiable ID.

Genetic analysis

We will extract total RNA from blood samples, and perform qPCR to quantify the expression of long non-coding RNA and associated mRNA. We aim to determine relative expression of candidate transcripts across the multiple patient phenotypes of coronary artery disease.

Data collection and record linkage:

CHI is a population register containing details of all Scottish residents registered with a General Practitioner and will be used to link all data sources. The ***Scottish Morbidity Record (SMR)*** will be used to identify the rate of myocardial infarction or cardiovascular death at 1 year. As in our previous studies, additional clinical information will be obtained through the TrakCare software application (InterSystems Corporation, Cambridge, MA, USA); with further information collected through a standardised *pro forma*.[8,9] This will include details of their presenting complaint, risk factors and past medical history including the following: time of onset of symptoms, time of hospitalisation, patient demographics (e.g. age and sex, cardiovascular risk factors, medical therapy on admission), GRACE score, heart rate, blood pressure, management in the Emergency Department, referral to cardiology, and discharge location. Any change to medical therapy will be extracted from the patients' standardised electronic discharge summary. Reports from diagnostic coronary angiography, percutaneous and surgical coronary revascularisation will be extracted from the ***TOMCAT database*** (Cardiovascular Information Management System, Philips Healthcare). This information is stored locally on NHS Lothian servers. The trial results will be reported in accordance with the CONSORT guidelines and, where possible, the clinical profile of non-recruited and ineligible patients will be recorded with Caldicott approval.

8. Statistical Analysis

Power calculations:

This exploratory analysis will determine the prevalence of coronary artery disease in patients with myocardial injury secondary to oxygen supply and demand imbalance. During our pilot study,[19] 1,126 consecutive patients were recruited over eight weeks. An adjudicated diagnosis of type 2 myocardial infarction or myocardial injury was made in 74 patients, and we therefore anticipate that 9 patients per week will meet the inclusion and exclusion criteria. We aim to recruit 100 patients as this is a feasible sample size, and will complete recruitment over 12-18 months. As this is exploratory analysis, no formal power calculations have been performed.

Statistical analysis:

We will report the prevalence of coronary artery stenosis in those with type 2 myocardial infarction. We will derive linear mixed-effects models to compare the release kinetics of cardiac troponin in patients with type 2 myocardial infarction, with a control cohort of individuals with type 1 myocardial infarction. We will determine whether differences in release kinetics may aid identification of such patients in clinical practice. We will validate a prediction model of coronary disease in patients with type 2 myocardial infarction, derived in the HighSTEACS clinical trial, with observed prevalence of coronary artery disease in the DEMAND-MI study.

9. Expected Results

To date, no study has prospectively evaluated patients with myocardial injury secondary to myocardial oxygen supply or demand imbalance for the presence of obstructive coronary artery disease. From our previous studies, we know patients with these diagnoses are older, and have a higher prevalence of co-morbid conditions such as hypertension and hyperlipidaemia. We anticipate that the majority of patients will have evidence of obstructive coronary artery disease on diagnostic testing. At present, this patient population are understudied and have extremely poor outcomes. By determining the role of coronary artery disease in the pathogenesis of type 2 myocardial infarction and myocardial injury, we can better inform clinicians on the importance of risk stratification for coronary disease in this population. Ultimately, this study will guide the rationale and design of future therapeutic trials of secondary prevention in patients with type 2 myocardial infarction and myocardial injury.

10. Safety Reporting and Study Monitoring

10.1 Trial Management Group

The trial management group will meet regularly and consists of the grant applicants, the trial manager, and research team.

10.2 Safety Reporting

An **adverse event** (AE) is any untoward medical occurrence in a study participant, which does not necessarily have a causal relationship with the study intervention.

An **adverse reaction** (AR) is any untoward and unintended response that has occurred due to the intervention.

A **serious adverse event** (SAE) or **serious adverse reaction** (SAR). Any AE or AR that:

- results in death of the study participant;
- is life threatening*;
- requires in-patient hospitalisation^ or prolongation of existing hospitalisation;
- results in persistent or significant disability or incapacity;
- consists of a congenital anomaly or birth defect;
- results in any other significant medical event not meeting the criteria above.

*Life-threatening in the definition of an SAE or SAR refers to an event where the participant was at risk of death at the time of the event. It does not refer to an event which hypothetically might have caused death if it were more severe.

^Any hospitalisation that was planned prior to enrolment will not meet SAE criteria. Any hospitalisation that is planned post enrolment will meet the SAE criteria.

10.3 identifying AEs and SAEs

The risk of AEs/SAEs relates to the investigations to be undertaken as part of the study, namely invasive coronary angiography, CT coronary angiography or cardiac magnetic resonance imaging. Any likely AE or SAE will be identified at or immediately after the time of the investigation taking place. Patients will be given the opportunity to contact the research team should they develop new symptoms. If there is any doubt as to whether a clinical observation is an AE, the event will be recorded.

10.4 Recording AEs and SAEs

When an AE/SAE occurs, the Investigator, or another suitably qualified physician in the research team will review all documentation (e.g. hospital notes, laboratory and diagnostic reports) related to the event. The Investigator will then record all relevant information in the Case Record Form and or AE log and on the SAE form (if the AE meets the criteria of serious). The information to be recorded will include the type of event, onset date, assessment of severity and causality, date of resolution and treatment required, investigations needed and outcomes. Pre-existing medical conditions (i.e. existed prior to informed consent) will be recorded as medical history and only recorded as adverse events if medically judged to have

worsened during the study. All AE or SAE will be recorded from the time of first intervention. AEs and SAEs will be followed up until outcome of recovered, recovered with sequelae or death of study participant.

We will not record, notify or report the development of a simple radial or femoral haematoma related to invasive coronary angiography where this is managed conservatively with pressure and where hospital admission was not required.

Worsening of the Underlying Condition during the Trial

Medical occurrences or symptoms of deterioration that are expected due to the participant's underlying condition will be recorded in the patient's medical notes and will only be recorded as AEs on the AE log if medically judged to have unexpectedly worsened during the study. Events that are consistent with the expected progression of the underlying disease will not be recorded as AEs.

10.5 Assessment of AEs and SAEs

Each AE will be assessed for seriousness, causality, severity and ARs will be assessed for expectedness by the Principal Investigator or another suitably qualified physician in the research team who has been delegated this role.

Assessment of Seriousness

The Investigator will make an assessment of seriousness as defined above.

Assessment of Causality

The Investigator will make an assessment of whether the AE/SAE is likely to be related to the study intervention according to the definitions below.

- Unrelated: where an event is not considered to have occurred as a result of the study intervention.
- Possibly Related: The nature of the event, the underlying medical condition, concomitant medication or temporal relationship make it possible that the AE has a causal relationship to the study intervention.

If two assessments of causality are made (for example between the Primary and the Chief Investigator), the 'worst case' assessment will be used for reporting purposes.

Assessment of Expectedness

If the AE is judged to be related to the study intervention, the Investigator will make an assessment of expectedness:

Expected: the type of event is expected in line with the study intervention

Unexpected: the type of event was not listed in the protocol or related documents/literature as an expected occurrence.

Assessment of Severity

The Investigator will make an assessment of severity for each AE and record this on the CRF/AE log or SAE form according to one of the following categories:

Mild: an event that is easily tolerated by the participant, causing minimal discomfort and not interfering with every day activities.

Moderate: an event that is sufficiently discomforting to interfere with normal everyday activities.

Severe: an event that prevents normal everyday activities.

10.6 Reporting of SAEs

*Once the Investigator becomes aware that an SAE has occurred in a study participant, the information will be reported to the ACCORD Research Governance **within 24 hours**. If the Investigator does not have all information regarding an SAE, they should not wait for this additional information before notifying ACCORD. The SAE report form can be updated when the additional information is received.*

The SAE report will provide an assessment of causality and expectedness at the time of the initial report to ACCORD according to Sections 10.5 Assessment of Causality and 10.5, Assessment of Expectedness.

The SAE form will be transmitted via email to safety@accord.scot

10.7 Follow up procedures

After initially recording an AE or recording and reporting an SAE, the Investigator will make every effort to follow each event until a final outcome can be recorded or reported as necessary. Follow up information on an SAE will be reported to the ACCORD office. If, after follow up, resolution of an event cannot be established, an explanation will be recorded on the CRF or AE log or additional information section of SAE form.

11. Oversight Arrangements

Inspection of Records

Investigators and institutions involved in the study will permit trial related monitoring and audits on behalf of the sponsor, REC review, and regulatory inspection(s). In the event of audit or monitoring, the Investigator agrees to allow the representatives of the sponsor direct access to all study records and source documentation. In the event of regulatory inspection, the Investigator agrees to allow inspectors direct access to all study records and source documentation.

Risk Assessment

A study specific risk assessment will be performed by representatives of the co-sponsors, ACCORD monitors and the QA group, in accordance with ACCORD governance and sponsorship SOPs. Input will be sought from the Chief Investigator or designee. The outcomes of the risk assessment will form the basis of the monitoring plans and audit plans. The risk assessment outcomes will also indicate which risk adaptions (delete if no adaptations were possible) could be incorporated into to trial design.

Study Monitoring And Audit

The ACCORD Sponsor Representative will assess the study to determine if an independent risk assessment is required. If required, the independent risk assessment will be carried out by the ACCORD Quality Assurance Group to determine if an audit should be performed before/during/after the study and, if so, at what frequency.

Risk assessment, if required, will determine if audit by the ACCORD QA group is required. Should audit be required, details will be captured in an audit plan. Audit of Investigator sites, study management activities and study collaborative units, facilities and 3rd parties may be performed.

12. Good Clinical Practice

Ethical Conduct

The study will be conducted in accordance with the principles of the International Conference on Harmonisation Tripartite Guideline for Good Clinical Practice (ICH GCP).
Before the study can commence, all required approvals will be obtained and any conditions of approvals will be met.

Investigator Responsibilities

The Investigator is responsible for the overall conduct of the study at the site and compliance with the protocol and any protocol amendments. In accordance with the principles of ICH GCP, the following areas listed in this section are also the responsibility of the Investigator. Responsibilities may be delegated to an appropriate member of study site staff.

Informed Consent

The Investigator is responsible for ensuring informed consent is obtained before any protocol specific procedures are carried out. The decision of a participant to participate in clinical research is voluntary and should be based on a clear understanding of what is involved.

Participants must receive adequate oral and written information – appropriate Participant Information and Informed Consent Forms will be provided. The oral explanation to the participant will be performed by the Investigator or qualified delegated person, and must cover all the elements specified in the Participant Information Sheet and Consent Form.

The participant must be given every opportunity to clarify any points they do not understand and, if necessary, ask for more information. The participant must be given sufficient time to consider the information provided. It should be emphasised that the participant may withdraw their consent to participate at any time without loss of benefits to which they otherwise would be entitled.

The participant will be informed and agree to their medical records being inspected by regulatory authorities and representatives of the sponsor(s).

The Investigator or delegated member of the trial team and the participant will sign and date the Informed Consent Form(s) to confirm that consent has been obtained. The participant will receive a copy of this document and a copy filed in the Investigator Site File (ISF) and participant’s medical notes (if applicable).

Study Site Staff

The Investigator must be familiar with the protocol and the study requirements. It is the Investigator's responsibility to ensure that all staff assisting with the study are adequately informed about the protocol and their trial related duties.

Data Recording

The Principal Investigator is responsible for the quality of the data recorded in the CRF at each Investigator Site.

Investigator Documentation

The Principal Investigator will ensure that the required documentation is available in local Investigator Site files ISFs.

GCP Training

For non-CTIMP (i.e. non-drug) studies all researchers are encouraged to undertake GCP training in order to understand the principles of GCP. However, this is not a mandatory requirement unless deemed so by the sponsor. GCP training status for all investigators should be indicated in their respective CVs.

Confidentiality

All laboratory specimens, evaluation forms, reports, and other records must be identified in a manner designed to maintain participant confidentiality. All records must be kept in a secure storage area with limited access. Clinical information will not be released without the written permission of the participant. The Investigator and study site staff involved with this study may not disclose or use for any purpose other than performance of the study, any data, record, or other unpublished, confidential information disclosed to those individuals for the purpose of the study. Prior written agreement from the sponsor or its designee must be obtained for the disclosure of any said confidential information to other parties.

Data Protection

All Investigators and study site staff involved with this study must comply with the requirements of the Data Protection Act 1998 with regard to the collection, storage, processing and disclosure of personal information and will uphold the Act's core principles. Access to collated participant data will be restricted to individuals from the research team treating the participants, representatives of the sponsor(s) and representatives of regulatory authorities.

Computers used to collate the data will have limited access measures via user names and passwords. Published results will not contain any personal data that could allow identification of individual participants.

13. Study Conduct Responsibilities

Protocol Amendments

Any changes in research activity, except those necessary to remove an apparent, immediate hazard to the participant in the case of an urgent safety measure, must be reviewed and approved by the Chief Investigator.

Amendments will be submitted to a sponsor representative for review and authorisation before being submitted in writing to the appropriate REC, and local R&D for approval prior To Participants Being Enrolled Into An Amended Protocol.

Management of Protocol Non Compliance

Prospective protocol deviations, i.e. protocol waivers, will not be approved by the sponsors and therefore will not be implemented, except where necessary to eliminate an immediate hazard to study participants. If this necessitates a subsequent protocol amendment, this should be submitted to the REC, and local R&D for review and approval if appropriate.

Protocol deviations will be recorded in a protocol deviation log and logs will be submitted to the sponsors every 3 months. Each protocol violation will be reported to the sponsor within 3 days of becoming aware of the violation. All protocol deviation logs and violation forms should be emailed to QA@accord.scot

Deviations and violations are non-compliance events discovered after the event has occurred. Deviation logs will be maintained for each site in multi-centre studies. An alternative frequency of deviation log submission to the sponsors may be agreed in writing with the sponsors.

Serious Breach Requirements

A serious breach is a breach which is likely to effect to a significant degree:

(a) the safety or physical or mental integrity of the participants of the trial; or

(b) the scientific value of the trial.

If a potential serious breach is identified by the Chief investigator, Principal Investigator or delegates, the co-sponsors (seriousbreach@accord.scot) must be notified within 24 hours. It is the responsibility of the co-sponsors to assess the impact of the breach on the scientific value of the trial, to determine whether the incident constitutes a serious breach and report to research ethics committees as necessary.

Study Record Retention

All study documentation will be kept for a minimum of 3 years from the protocol defined end of study point. When the minimum retention period has elapsed, study documentation will not be destroyed without permission from the sponsor.

End of Study

The end of study is defined as the last participant's last visit.

The Investigators or the co-sponsor(s) have the right at any time to terminate the study for clinical or administrative reasons.

The end of the study will be reported to the REC, and R+D Office(s) and co-sponsors within 90 days, or 15 days if the study is terminated prematurely. The Investigators will inform participants of the premature study closure and ensure that the appropriate follow up is arranged for all participants involved. End of study notification will be reported to the co-sponsors via email to resgov@accord.scot.

A summary report of the study will be provided to the REC within 1 year of the end of the study.

Continuation of treatment following the end of study

Detail if intervention will be continued to be provided following the end of the study. If not provide justification

Insurance and Indemnity

The co-sponsors are responsible for ensuring proper provision has been made for insurance or indemnity to cover their liability and the liability of the Chief Investigator and staff.

The following arrangements are in place to fulfil the co-sponsors' responsibilities:

- The Protocol has been designed by the Chief Investigator and researchers employed by the University and collaborators. The University has insurance in place (which includes no-fault compensation) for negligent harm caused by poor protocol design by the Chief Investigator and researchers employed by the University.
- Sites participating in the study will be liable for clinical negligence and other negligent harm to individuals taking part in the study and covered by the duty of care owed to them by the sites concerned. The co-sponsors require individual sites participating in the study to arrange for their own insurance or indemnity in respect of these liabilities.
- Sites which are part of the United Kingdom's National Health Service will have the benefit of NHS Indemnity.
- Sites out with the United Kingdom will be responsible for arranging their own indemnity or insurance for their participation in the study, as well as for compliance with local law applicable to their participation in the study.

14. References

1. Thygesen K, Alpert JS, Jaffe AS, Simoons ML, Chaitman BR, White HD. Third universal definition of myocardial infarction. *Eur Heart J.* 2012; 33: 2551-2567.
2. Morrow DA, Wiviott SD, White HD, Nicolau JC, Bramucci E, Murphy SA, Bonaca MP, Ruff CT, Scirica BM, McCabe CH, Antman EM, Braunwald E. Effect of the novel thienopyridine prasugrel compared with clopidogrel on spontaneous and procedural myocardial infarction in the trial to assess improvement in therapeutic outcomes by optimizing platelet inhibition with prasugrel-thrombolysis in myocardial infarction 38: an application of the classification system from the universal definition of myocardial infarction. *Circulation*. 2009;119(21):2758-64.
3. Bonaca MP, Wiviott SD, Braunwald E, Murphy SA, Ruff CT, Antman EM, Morrow DA. American College of Cardiology/American Heart Association/European Society of Cardiology/World Heart Federation universal definition of myocardial infarction classification system and the risk of cardiovascular death: observations from the TRITON-TIMI 38 trial. *Circulation*. 2012;125(4):577-83.
4. White HD, Reynolds HR, Carvalho AC, Pearte CA, Liu L, Martin E, Knatterud GL, Džavík V, Kruk M, Steg PG, Cantor WJ, Menon V, Lamas GA, Hockman JS. Reinfarction after percutaneous coronary intervention or medical management using the universal definition in patients with total occlusion after myocardial infarction: Results from long-term follow-up of the occluded artery trial (OAT) cohort. *Am Heart J*. 2012;163:563-571.
5. Alpert JS, Thygesen KA. The Case for a Revised Definition of Myocardial Infarction – The Ongoing Conundrum of Type 2 Myocardial Infarction vs Myocardial Injury. *JAMA Cardiol.* 2016;1(3):249-250
6. Alpert JS, Thygesen KA, White HD, Jaffe AS. Diagnostic and therapeutic implications of type 2 myocardial infarction: review and commentary. *Am J Med*. 2014;127(2):105-8.
7. Sandoval Y, Smith SW, Thordsen SE, Apple FS. Supply/demand type 2 myocardial infarction: Should we be paying more attention? *J Am Coll Cardiol.* 2014;63(20):2079-2087.
8. Mills NL, Churchhouse AM, Lee KK, Anand A, Gamble G, Shah AS, Paterson E, MacLeod M, Graham G, Walker S, Denvir MA, Fox KA, Newby DE. Implementation of a sensitive troponin I assay and risk of recurrent myocardial infarction and death in patients with suspected acute coronary syndrome. *JAMA*. 2011;305:1210-16.
9. Mills NL, Lee KK, McAllister DA, Churchhouse AM, MacLeod M, Stoddart M, Walker S, Denvir MA, Fox KA, Newby DE. Implications of lowering threshold of plasma troponin concentration in diagnosis of myocardial infarction: cohort study. *BMJ*. 2012;344:e1533
10. Shah AS, McAllister DA, Mills R, Lee KK, Churchhouse AM, Fleming KM, Layden E, Anand A, Fersia O, Joshi NV, Walker S, Jaffe AS, Fox KA, Newby DE, Mills NL. Sensitive

troponin assay and the classification of myocardial infarction. *Am J Med.* 2015;128(5):493-501.
11. Javed U, Aftab W, Ambrose JA, Wessel RJ, Mouanoutoua M, Huang G, Barua RS, Weilert M, Sy F, Thatai D. Frequency of elevated troponin I and diagnosis of acute myocardial infarction. *Am J Cardiol*. 2009;104(1):9-13.
12. Saaby L, Poulsen TS, Hosbond S, Larsen TB, Pyndt Diederichsen AC, Hallas J, Thygesen K, Mickley H. Classification of myocardial infarction: frequency and features of type 2 myocardial infarction. *Am J Med*. 2013;126(9):789-97.
13. Szymański FM, Karpiński G, Płatek AE, Majstrak F, Hrynkiewicz-Szymańska A, Kotkowski M, Puchalski B, Filipiak KJ, Opolski G. Clinical characteristics, aetiology and occurrence of type 2 acute myocardial infarction. *Kardiol Pol*. 2014;72(4):339-44.
14. Stein GY, Herscovici G, Korenfeld R, Matetzky S, Gottlieb S, Alon D, Gevrielov-Yusim N, Iakobishvili Z, Fuchs S. Type-II myocardial infarction--patient characteristics, management and outcomes. *PLoS One*. 2014;9:e84285.
15. Baron T, Hambraeus K, Sundstrom J, Erlinge D, Jernberg T, Lindahl B, TOTAL-AMI study group. Type 2 myocardial infarction in clinical practice. *Heart*. 2015;101:101–106. doi:10.1136/heartjnl-2014-306093
16. Gudmundsdottir I, Adamson P, Gray C, Spratt JC, Behan MW, Henriksen P, Newby DE, Mills N, Uren NG, Cruden NL. Optical coherence tomography versus intravascular ultrasound to evaluate stent implantation in patients with calcific coronary artery disease. *Open Heart* 2015;2.
17. Newby DE on behalf of the SCOT-HEART Investigators. CT coronary angiography in patients with suspected angina due to coronary artery disease (SCOT-HEART): an open-label, parallel-group, multicentre trial. Lancet 2015; 385(2383-2391).
18. Min JK, Leipsic J, Pencina MJ, Berman DS, Koo B-K, van Mieghem C et al. Diagnostic Accuracy of Fractional Flow Reserve From Anatomic CT Angiography. JAMA 2012; 308(12):1237-1245.
19. Shah AS, Griffiths M, Lee KK, McAllister DA, Hunter AL, Ferry AV, Cruikshank A, Reid A, Stoddart M, Strachan F, Walker S, Collinson PO, Apple FS, Gray AJ, Fox KA, Newby DE, Mills NL. High sensitivity cardiac troponin and the under-diagnosis of myocardial infarction in women: prospective cohort study. *BMJ* 2015;350:g7873
20. Chin CL, Shah AS, McAllister DA, Cowell JS, Alam S, Langrish JP, Strachan F, Hunter A, Choy AM, Lang CC, Walker S, Boon NA, Newby DE, Mills NL, Dweck MR. High-sensitivity troponin I concentrations are a marker of an advanced hypertrophic response and adverse outcomes in patients with aortic stenosis. *Eur Heart J.* 2014;35(34):2312-21.
